# Production of Acetaldehyde via Oxidative Dehydrogenation of Ethanol in a Chemical Looping Setup

**DOI:** 10.1021/acsengineeringau.2c00052

**Published:** 2023-02-28

**Authors:** Joseph C. Gebers, Abu Farhan Bin Abu Kasim, George J. Fulham, Kien Yi Kwong, Ewa J. Marek

**Affiliations:** Department of Chemical Engineering and Biotechnology, University of Cambridge, Philippa Fawcett Drive, Cambridge CB3 0AS, United Kingdom

**Keywords:** oxidative dehydrogenation, chemical looping, SrFeO_3_, acetaldehyde, silver

## Abstract

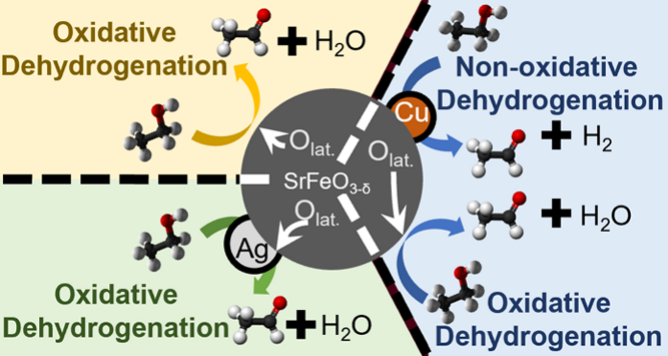

A novel chemical looping (CL) process was demonstrated to produce acetaldehyde (AA) via oxidative dehydrogenation (ODH) of ethanol. Here, the ODH of ethanol takes place in the absence of a gaseous oxygen stream; instead, oxygen is supplied from a metal oxide, an active support for an ODH catalyst. The support material reduces as the reaction takes place and needs to be regenerated in air in a separate step, resulting in a CL process. Here, strontium ferrite perovskite (SrFeO_3−δ_) was used as the active support, with both silver and copper as the ODH catalysts. The performance of Ag/SrFeO_3−δ_ and Cu/SrFeO_3−δ_ was investigated in a packed bed reactor, operated at temperatures from 200 to 270 ^°^C and a gas hourly space velocity of 9600 h^–1^. The CL capability to produce AA was then compared to the performance of bare SrFeO_3−δ_ (no catalysts) and materials comprising a catalyst on an inert support, Cu or Ag on Al_2_O_3_. The Ag/Al_2_O_3_ catalyst was completely inactive in the absence of air, confirming that oxygen supplied from the support is required to oxidize ethanol to AA and water, while Cu/Al_2_O_3_ gradually got covered in coke, indicating cracking of ethanol. The bare SrFeO_3−δ_ achieved a similar selectivity to AA as Ag/SrFeO_3−δ_ but at a greatly reduced activity. For the best performing catalyst, Ag/SrFeO_3−δ_, the obtained selectivity to AA reached 92–98% at yields of up to 70%, comparable to the incumbent Veba-Chemie process for ethanol ODH, but at around 250 ^°^C lower temperature. The CL-ODH setup was operated at high effective production times (i.e., the time spent producing AA to the time spent regenerating SrFeO_3−δ_). In the investigated configuration with 2 g of the CLC catalyst and 200 mL/min feed flowrate ∼5.8 vol % ethanol, only three reactors would be required for the pseudo-continuous production of AA via CL-ODH.

## 1 Introduction

Acetaldehyde (AA, CH_3_CHO) is a versatile chemical intermediate used to synthesize acetic acid, peracetic acid, pyridine bases, and other chemicals.^1^ Commercially, AA is produced by (1) dehydrogenation or oxidative dehydrogenation of ethanol (C_2_H_5_OH), (2) addition of water to acetylene, (3) direct oxidation of ethylene, or (4) partial oxidation of hydrocarbons. Only the first three routes are used industrially because method (4), partial oxidation, produces a mixture of products, giving relatively low selectivity to AA. Route (2) is attractive only when acetylene is more available than other raw materials, for example, alongside other industrial reactions in petrochemistry. In Europe, route (3), with direct oxidation of ethylene via the Wacker–Hoechst process, dominates because of the lower prices of ethylene compared to ethanol or acetylene. The Wacker–Hoechst process (100–130 °C, 4–10 bar) passes ethylene and air or purified O_2(g)_ through an acidic catalytic solution of PdCl_2_ and CuCl_2_, achieving 95% yields of AA.^1^ Although economically attractive, because the use of Pd is minimal, the Wacker–Hoechst oxidation suffers from corrosion issues, produces waste streams with chlorinated hydrocarbons, and uses non-renewable ethylene. The three problems make route (3), AA via ethylene, suitable only at an industrial scale.

In route (1), production of AA from ethanol involves dehydrogenation (R1) or oxidative dehydrogenation (R2) reactions:
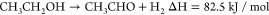
R1
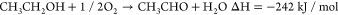
R2

The usual catalysts for ethanol dehydrogenation are Cu, Pt, and Cr, among other transition and rare earth metals,^2,3^ while Ag, Au, and V are applied in oxidative dehydrogenation^4−6^

Historically, both dehydrogenation processes have been implemented industrially, but the oxidative route via (R2) has been preferred because of the longer catalyst lifetime and heat production.^1^ One of the oldest methods is the Veba-Chemie process, carried out at 500–650 °C, by passing a mixture of ethanol and air through a bed of catalyst, typically reaching 50–70% alcohol conversion and 97–99% selectivity.^1^ However, the oxidative route requires careful temperature control to avoid over-oxidation of AA to CO_2_. Another issue concerns the safety of mixing O_2_ with ethanol^7^ with flammable mixtures forming at ethanol concentrations of 3.3–19 vol % in air. Thus, the oxidative route with ethanol is, similar to route (3) with ethylene, feasible only at a large scale and after careful heat integration.

Non-oxidative dehydrogenation of ethanol (R1) has been gaining interest because of the co-production of hydrogen and the lower operating temperature range, 200–300°C.^8^ The endothermic non-oxidative route is thermodynamically favored only above 600 K, with low-temperature reactions being strongly limited with respect to achievable conversion.^9^ Thus, as explained by Garbarino et al.,^9^ a highly selective dehydrogenation catalyst is required, and inexpensive, copper-based catalysts are preferred, but because Cu-catalysts tend to sinter and coke,^10^ most research has focused on improving their lifetime.^2,11^ Zhang et al. reached over 90% conversion of ethanol and 98% selectivity to AA, running the reaction for 500 h with an engineered catalyst comprising Cu nanoparticles (∼3 nm) on SiO_2_.^12^ However, slow but still noticeable sintering of Cu and the low hourly spaced velocity (HSV) hinder a confident prediction of the performance at an industrial scale. From a similar study but with high HSV, Liu et al. reported that well-dispersed Cu nanoparticles started agglomerating after 150 h on stream.^8^

This study proposes a novel route to produce AA from ethanol, employing a chemical looping (CL) approach, with a catalyst for dehydrogenation deposited on a metal oxide capable of donating oxygen to the reaction:
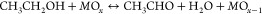
R3a

The metal oxide is then regenerated by reoxidation in air in a separate step:
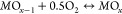
R3b

CL offers increased safety in oxidative catalytic reactions, removing the need to mix O_2(g)_ with flammable reactants and eliminating the problem of costly separation of depleted air from the products. Originally proposed for combustion and effective CO_2_ capture, CL has been shown to be effective and selective in catalytic processes, for example, epoxidation of olefins^13^ and oxidative dehydration of alkanes.^14,15^ CL for oxidative dehydrogenation of ethanol has been suggested by Zhu et al. in their recent perspective paper,^16^ but until now, the process has not been demonstrated experimentally.

Here, SrFeO_3_ perovskite was employed as *M*O_*x*_ because SrFeO_3_ has been shown to be an effective donor of oxygen during CL epoxidation of ethylene at 270 °C.^17^ For the production of AA, Ag and Cu catalysts were used after depositing them on SrFeO_3_. The catalysts were also deposited on Al_2_O_3_, which is incapable of donating oxygen at low temperatures. When running the oxidative dehydrogenation in the CL mode, only AA was the main product because H_2_ was oxidized to water (R3a). Yet, the presence of lattice oxygen from *M*O_*x*_ can be beneficial in minimizing coke deposition, helping to avoid deactivation seen in the non-oxidative dehydrogenation. CL also allows operating at low temperatures, below 300 °C, and with enhanced safety, thus improving on the incumbent oxidative Veba-Chemie process.

## 2 Experimental Section

### 2.1 Materials

Strontium ferrite (SrFeO_3_) was prepared by a solid-state synthesis method described in an earlier study.^17^ Shortly, stoichiometric amounts of SrCO_3_ (Sigma Aldrich, ≥98%) and Fe_2_O_3_ (Honeywell, ≥99%) were mixed with ethanol (50 mL, 99.8%, Fisher Scientific) and then ball-milled for 30 min at 600 rpm. After ball-milling, the mixture was dried for 24 h at 50 °C and sieved to 180–355 μm. The material was then calcined four times for 3 h in static air at 1000 °C, with a ramp rate of 5 °C min^–1^, and again sieved to 180–355 μm. The obtained oxide has a stoichiometry of SrFeO_2.82_^18^ but will be denoted as SrFeO_3_.

For an inert support material, incapable of donating oxygen at low temperatures, Al_2_O_3_ was chosen. Particles of α-Al_2_O_3_ (Boud Minerals) were crushed and sieved to 355–425 μm.

Impregnation of Ag onto the surface of SrFeO_3_ or Al_2_O_3_ was done by incipient wetness impregnation. First, AgNO_3_ (Alfa Aesar, ≥99.9%) or Cu(NO_3_)_2_·2.5H_2_O (Sigma Aldrich, 98%) was dissolved in deionized water. The volume of the prepared solution was adjusted to match the accessible pore volume of SrFeO_3_ or Al_2_O_3_ particles, determined empirically beforehand. Then, the prepared nitrate solution was added dropwise to a 5.00 g batch of SrFeO_3_ or Al_2_O_3_ particles, while mixing the particles with a spatula. The sample was dried in static air at 120 °C for 12 h, followed by calcination at 550–650 °C for 5 h. Nominal metal loadings of Ag and Cu were 15 wt %, except for Cu on SrFeO_3_, which was 10 wt %. The loading for this last sample was lower than for Ag, corresponding to the lower solubility of Cu(NO_3_)_2_ and lower possible mass of this precursor per fixed amount of water in impregnation. The high loading of Cu on Al_2_O_3_ was obtained by repeating the impregnation step. The Cu/Al_2_O_3_ particles were then calcined for the third time for 1 h at 800 °C.

### 2.2 Material Characterization

A Bruker D8 Advance diffractometer using Cu-Kα radiation (40 kV and 40 mA) was used to take powder X-ray diffraction (XRD) measurements on fresh and spent samples of SrFeO_3_, Ag/SrFeO_3_, and Cu/SrFeO_3_. Diffraction patterns were obtained with scans of 2θ ranging from 5° to 80°, with a step size of 0.05°. The resulting XRD patterns were analyzed with Profex^19^ to quantify the phase composition. The following reference patterns were used: ICSD-91062 (SrFeO_3_), ICSD-51318 (SrFeO_2.5_), ICSD-75522 (Sr_3_Fe_2_O_7_), ICSD-69022 (SrFe_12_O_19_), ICSD-15195 (SrCO_3_), ICSD-105548 (SrO), ICSD-52545 (Ag), ICSD-7945 (Cu), ICSD-16025 (CuO), and ICSD-54126 (Cu_2_O).

The mean crystallite sizes of Ag, Cu, and CuO in fresh and spent samples of Ag/SrFeO_3_ and CuO/SrFeO_3_ were determined using the Scherrer equation (eq 1)
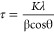
1where τ is the mean crystallite size (nm), *Κ* is a dimensionless shape factor (taken to be 0.9), λ is the X-ray wavelength (0.15406 nm), β is the full width at half maximum of a peak (radians), and θ is the Bragg angle (radians).

The Cu and Ag content in fresh and spent samples of CuO/SrFeO_3_ and Ag/SrFeO_3_ was measured with inductively coupled plasma atomic emission spectroscopy (ICP-AES). The Al content in the spent samples was also measured to account for possible contamination of the spent samples with Al_2_O_3_.

Bright-field (BF) and high-angle annular dark field scanning transmission electron microscopy (STEM) images and energy-dispersive X-ray analysis (EDS) maps were acquired using a Thermo Scientific (FEI) Talos F200X G2 operating at 200 kV. STEM images were collected at a camera length of 98 mm and EDS maps using the Super-X EDS detector system, which consists of four windowless silicon drift detectors. The samples were prepared by pipetting 3 μL of an isopropyl alcohol suspension onto holey carbon film on 300 mesh Cu/Ni grids (EM Resolutions). The size distributions of the metallic particles were estimated by manual measurements of the diameter of Ag, Cu, and CuO particles from BF-STEM images overlayed with Cu or Ag EDS maps using ImageJ software.^20^ Histograms showing the particle size distributions are shown in Figure S3.

The specific surface area, pore volume, and pore size of fresh and spent samples of SrFeO_3_, Ag/SrFeO_3_, and CuO/SrFeO_3_ were determined from N_2_ adsorption–desorption isotherms at 77 K, collected with a Micromeritics 3Flex instrument, with the dewar filled with liquid N_2_. Prior to analysis, samples were degassed overnight under vacuum (Micromeritics VacPrep) at 100 °C. The specific surface area was calculated using the Brunauer–Emmett–Teller (BET) Surface Identification (BETSI) software,^21^ and the specific pore volume and pore size were calculated from Barrett–Joyner–Halenda (BJH) analysis.

The extent of carbonation and coke deposition on the spent samples of SrFeO_3_, Ag/SrFeO_3_, and Cu/SrFeO_3_ was evaluated using thermogravimetric analysis (TGA) with a TGA/DSC 1, Mettler Toledo. Approximately 40 mg of a sample was introduced into a 70 μL alumina crucible and placed on the TGA instrument’s balance. Three types of experiments were carried out, all consisting of two to four temperature-programmed cycles with the TGA chamber heated from 50 to 900 °C and cooled back down to 50 °C, all at a temperature ramp rate of 10 °C min^–1^. The runs differed in the applied “reactive” gas that were passed over the sample at 50 mL min^–1^ (NTP) via a capillary tube positioned above the crucible. The three types of experiments were as follows: (1) two temperature cycles in blended air (21 ± 0.5 vol % O_2_, balance N_2_, BOC); (2) two temperature cycles in CO_2_ (99.8 vol %, BOC), followed by two cycles in blended air; (3) one temperature cycle in N_2_ (99.99 vol %, BOC), followed by one cycle in blended air. The TGA chamber was purged with 100 mL min^–1^ (NTP) of N_2_ throughout the experiments; thus, the effective O_2_ and CO_2_ concentrations were 7 and 33.3 vol %, respectively. A blank experiment was performed using 40 mg of silica sand, and the result was extracted from the values recorded with the reactive samples, thus removing the influence of the buoyancy effects. The recorded masses were normalized against the sample mass recorded at the end of the first cycle in air to ensure that the 100 wt % relative mass was of a re-oxidized sample, free of coke and carbonates. A second set of TGA experiments was conducted with isothermal gas switching at 250 °C, with a gas program that followed the CL experiments: (1) 10 min of N_2_ flow, (2) 30 min of ∼4 vol % ethanol in N_2_; (3) 10 min of N_2_ flow; and (4) 120 min of air flow. Prior to the isothermal experiments, the samples were cleaned by heating under air flow to 900 °C at 10 °C min^–1^ and subsequent cooling to 250 °C.

Temperature-programmed reduction (H_2_-TPR) was performed under hydrogen, using a Micromeritics Autochem II 2920. Prior to each experiment, 50 mg of the sample was cleaned at 650 °C for 5 h, followed by cooling down to ambient temperature, under 25 mL min^–1^ (STP) air flow. The gas supply was then switched to H_2_ (5 vol % in N_2_) at 25 mL min^–1^ (STP), and the sample was heated from 50 to 900 °C with a 10 °C min^–1^ heating rate. The outlet gas was passed through a cold trap of liquid nitrogen and isopropanol to remove water. The dried gas was then passed to an on-line thermal conductivity detector (TCD) to measure H_2_ and on-line MKS Cirrus mass spectrometer.

### 2.3 Experiments in a Packed Bed

**Figure 1:**
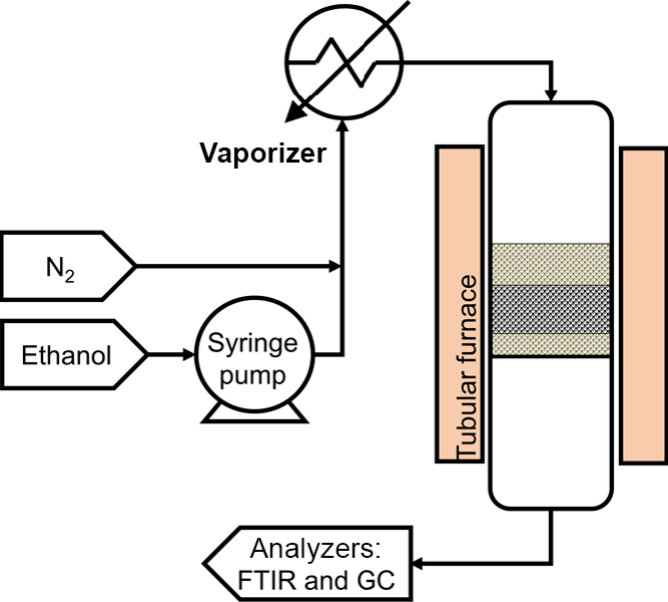
Schematic representation of the experimental setup in packed-bed configuration.

Experiments in a packed bed were performed in a reactor made of a stainless-steel tube, 15 mm i.d., placed in an electric furnace. The rig is schematically depicted in Figure 1. The packed bed was created comprising three layers: (1) a bottom layer of 1 g α-Al_2_O_3_ (300–425 μm); (2) a middle layer of 2 g catalyst; and (3) a top layer of 4 g α-Al_2_O_3_. Gas was introduced from the top of the bed; hence, the last layer helped with gas preheating. A K-type thermocouple was placed in the middle of the catalyst bed to measure the temperature and control electrical heating, with the setpoint temperature varying between 200 and 300 °C. The gas feed used in experiments was prepared by passing 188 mL min^–1^ N_2_ (99.99 vol %, BOC) through an electrically heated evaporator of ethanol, to which liquid ethanol was delivered at 33 μL/min using a syringe pump. The resulting gas stream contained ∼5.8 vol % ethanol in N_2_, delivered at 200 mL min^–1^.

Experiments were carried out in a CL mode, meaning gases were provided to the reactor in a sequence that comprised (1) a reduction step using the ethanol/N_2_ stream, (2) purge in N_2_ for 2 min, (3) an oxidation step in blended air, and (4) purge in N_2_ for 2 min. Then, the sequence was repeated, as in CL cycles. The duration of step (1) varied from 1.5 to 60 min and step (3) from 1.5 to 15 min.

To analyze the content of H_2_ in the outlet gas after the packed bed reactor, a 20 mL sample of the outlet gas was collected manually and introduced into an Agilent 7890A gas chromatograph (GC), equipped with a Hayesep-Q/MolSieve 13A column and TCD and flame ionization detector (FID). The sample was drawn into the GC sample loop using a vacuum pump to ensure uniformity between sample injection volumes.

Gas from the packed bed reactor was passed continuously to a Fourier transform infrared (FTIR) analyzer (MKS Instruments, Multigas 2030), equipped with a liquid N_2_-cooled HgCdTe detector. Before each CL experiment, the detector was allowed to cool for 2 h; during this time, the packed bed was heated to 270 °C and kept at this temperature under a flow of air. A single FTIR scan was performed for 0.97 or 1.87 s, measuring the signal in the range of 100–5000 cm^–1^ at a resolution of 0.5 cm^–1^. The collected FTIR spectra were analyzed for ethanol, AA, CO, CO_2_, and H_2_O using MKS MG2000 software. The presence of other species was checked by running samples of the outlet gas in a GC; only small amounts of ethylene and ethyl acetate were detected, confirming that AA was the main hydrocarbon product from all experiments. The carbon balance was only closed for experiments (>95%) using bare SrFeO_3_; experiments with Ag/SrFeO_3_ had a carbon balance between 90 and 100%, while experiments with Cu/SrFeO_3_ had a carbon balance of 80–90%. The unclosed carbon balance confirms that some carbon-containing species (coke or carbonates) formed and deposited over Cu/SrFeO_3_ and to a lesser extent Ag/SrFeO_3_ during CL experiments.

Performance in reactions was assessed with selectivity for AA, *S*, and conversion of ethanol, *X*:
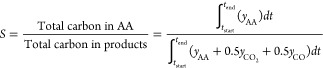
2where *t*_start_ is the point in each cycle where the concentration of AA started to exceed 50 ppm, *t*_end_ is the point where the measured concentration of ethanol started to decrease, and *y*_AA_, *y*_CO_2__, and *y*_CO_ are the measured concentrations of AA, CO_2_, and CO in ppm, respectively.
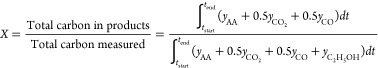
3where *y*_C_2_H_5_OH_ is the measured concentration of ethanol.

Then, the yield of produced AA *Y* is:
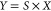
4

## 3 Results and Discussion

**Figure 2:**
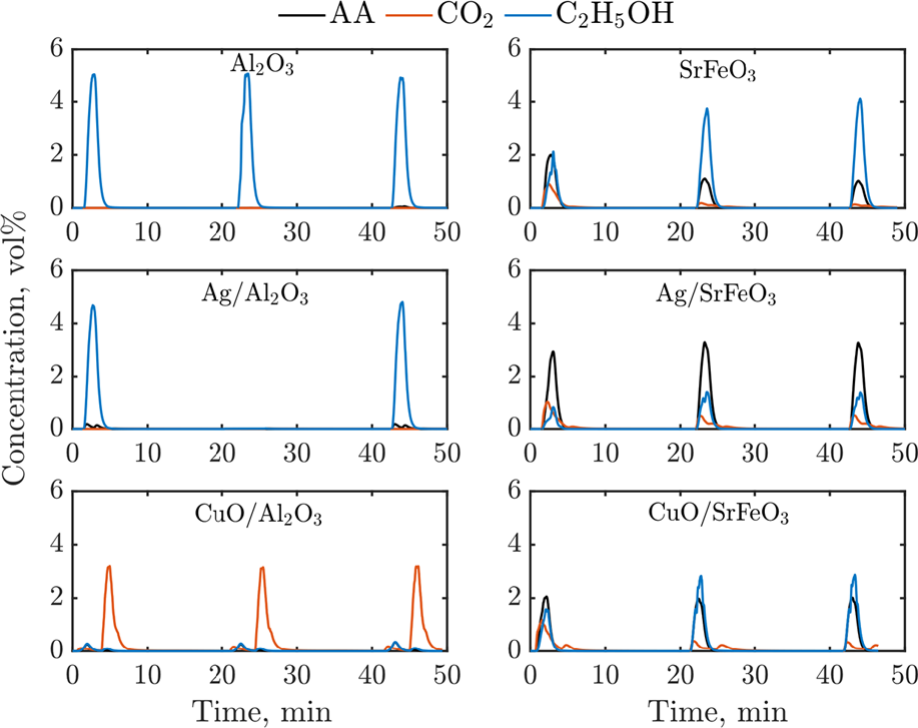
Ethanol, AA, and CO_2_ profiles during chemical looping experiments in a packed bed arrangement at 250 °C, comprising three chemical looping cycles of (1) 1.5 min reduction in 5.8 vol % ethanol/N_2_, (2) 2 min purge in N_2_, (3) 15 min reoxidation in air, and (4) 2 min purge in N_2_. A communication error appeared during the experiments with Ag/Al_2_O_3_; hence, the second chemical looping cycle was not recorded.

In total, six samples were investigated in experiments in the packed bed reactor. Those can be divided into materials with an active support, SrFeO_3_, Ag/SrFeO_3_, and CuO/SrFeO_3_, and materials with an inert support, Al_2_O_3_, Ag/Al_2_O_3_, and CuO/Al_2_O_3_. Results from experiments in CL mode when each bed was exposed to the flow of ethanol for 1.5 min and then regenerated in air for 15 min are presented in Figure 2.

The two first samples with alumina, Al_2_O_3_ alone and Ag/Al_2_O_3_, were practically inactive, with only small amounts of AA produced when Ag was present. The last sample on alumina, CuO/Al_2_O_3_, gave a small amount of CO_2_ during the reduction step, although most of the ethanol provided to the bed was consumed. The large release of CO_2_ during the reoxidation stage indicates that after the initial combustion of ethanol to CO_2_, which occurred using oxygen from CuO, ethanol must have decomposed on the resulting metallic Cu, depositing coke and releasing H_2(g)_. Indeed, as presented in Figure S1, the GC results confirmed that H_2_ and CO_2_ were the only major species generated in the reduction step. Interestingly, the behavior of this sample was repeatable from cycle to cycle, that is, only little CO_2_ was observed during the reduction step, and a significant amount of CO_2_ was released in the reoxidation step. Upon reoxidation at 250 °C, Cu did not regenerate to CuO because with the slow ionic diffusion of Cu^+^ at low temperatures, the rate of oxidation of Cu slows down after rapid surface passivation.^22^ Yabuki and Tanaka^23^ found that 20 nm Cu nanoparticles, similar as seen here, reoxidized to a Cu_2_O (80 wt %) core and CuO shell (20 wt %) after 5 min of oxidation in air at 240 °C.

Here, the combustion of ethanol was ascribed to CuO and Cu_2_O,^24^ while the cyclic catalytic cracking to Cu/Al_2_O_3_. As mentioned earlier, metallic copper is a catalyst commonly proposed for non-oxidative dehydrogenation of ethanol to H_2_ and AA; thus, the observed immediate coking was somewhat unexpected. Thus, prolonged reduction experiments were conducted, without reoxidation, to check whether Cu/Al_2_O_3_ becomes active after the first coking episode. Indeed, as presented in Figure S1, after 10 min of exposure to ethanol, the main reaction products confirm dehydrogenation, that is, Cu became selective toward AA and H_2_.

Overall, none of the samples containing Al_2_O_3_ resulted in a selective CL process, which would be associated with the release of oxygen from the solid carrier. The CuO-containing sample was active, but, instead of oxidative dehydration of ethanol, led to the catalytic decomposition of ethanol.

All samples with SrFeO_3_ produced noticeable amounts of AA (Figure 2). The FID results of GC measurements taken during the third CL over SrFeO_3_, Ag/SrFeO_3_, and CuO/SrFeO_3_ are shown in Figure S2 and confirm that AA and ethanol were the only hydrocarbon species detected at significant concentrations in the outlet gas. Because none of the investigated samples were pre-reduced, the fresh catalyst with copper contained CuO; however, the deposited oxide reduced in the first CL cycle, during which the donation of oxygen from CuO promoted the total combustion of ethanol to CO_2_. Thanks to the slow reoxidation of Cu, the behavior observed in the following cycles can be primarily ascribed to Cu_2_O/SrFeO_3_ and Cu/SrFeO_3_ (very little Cu components were detected in the reoxidized sample; see XRD results in Figure 9). The co-existence of Cu^+^ and Cu^0^ has been found to be synergistic for AA production before.^25^ Since CuO was not regenerated during the reoxidation step, the Cu-containing samples upon CL cycles are further referred to as Cu/SrFeO_3_ and Cu/Al_2_O_3_.

Among the three samples with SrFeO_3_, SrFeO_3_ alone was the least active, while the presence of a catalyst, Cu or Ag, enhanced the conversion of ethanol, with the main product remaining to be AA. The copper-containing sample supported catalytic cracking of ethanol similar to Cu/Al_2_O_3_, with coke combusting to CO_2_ during the reoxidation step. Although similar to the behavior of Cu/Al_2_O_3_, the extent of coking on Cu/SrFeO_3_ was significantly reduced, with SrFeO_3_ providing oxygen primarily to the ODH reaction. In-situ removal of coke using lattice oxygen from SrFeO_3_ can be ruled out because most ethanol ended up as AA rather than CO_2_.

**Figure 3:**
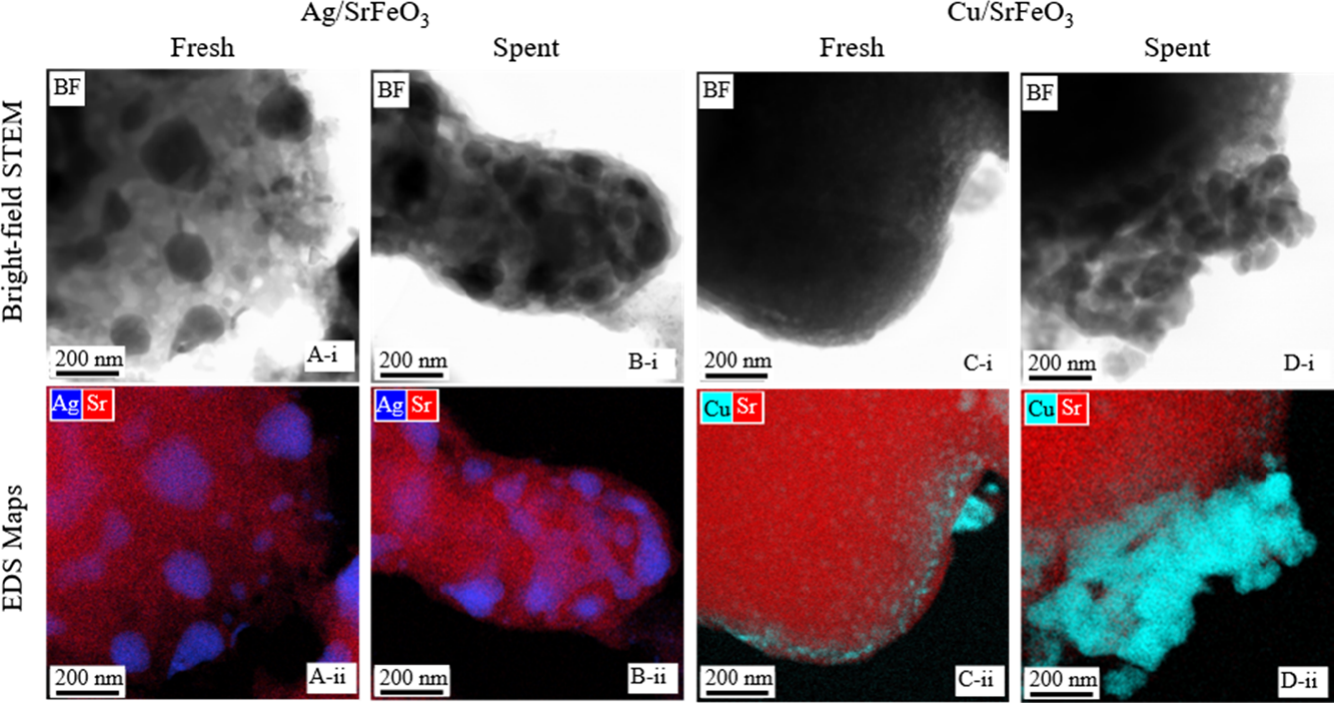
BF-STEM images (i) and STEM-EDS maps (ii) of crushed particles of (A) fresh Ag/SrFeO_3_; (B) spent Ag/SrFeO_3_; (C) fresh CuO/SrFeO_3_; and (D) spent Cu/SrFeO_3_ particles. The STEM-EDS maps show the distributions of Sr and Cu or Ag.

The STEM images and EDS maps are shown in Figure 3, and the corresponding mean particle size reported in Table 1 shows that the Ag in Ag/SrFeO_3_ existed as discrete nanoparticles in both spent and fresh samples. The mean sizes of the Ag particles of the fresh and spent samples were within a standard deviation from one another. The Ag content did not vary significantly between fresh and spent samples, determined from XRD Rietveld and ICP-AES analysis, which agrees with previous observations of Ag in CL experiments^26^ and the low *p*Ag at 250 °C.^27^ The presentation of Cu particles changed significantly during the CL experiments, as expected, because of (i) volumetric changes in CuO reduced to Cu and (ii) the well-known mobility of Cu particles that leads to sintering.^10^ The CuO particles in fresh CuO/SrFeO_3_ existed as discrete nanoparticles with a mean diameter of 71 ± 20 nm. Large clusters of Cu were created during dehydrogenation increasing the mean diameter to 234 ± 71 nm. The copper content, from ICP-AES, dropped following the CL experiments. This can be connected to the clustering of Cu and significant mobility of Cu particles under reaction, which might have resulted in particles with lowered or increased Cu content. The surface area of all samples was low, with BET surface areas of 1–4 m^2^ g^–1^, consistent with the surface areas reported on SrFeO_3_ prepared with a similar solid-state method.^13,26^ The surface areas reported by BET analysis are at a lower range of the detection limit for standard BET analysers,^28^ and thus, contribute to greater measurement uncertainty.

**Table 1:** Characterization of Fresh and Spent Samples of SrFeO_3_, Ag/SrFeO_3_, and Cu/SrFeO_3_ with ICP-OES, XRD, TEM, BJH, and BET Analysis^a^

analytical method		metallic content (wt %)		mean metallic particle size (nm)	mean metallic crystallite size (nm)	surface area (m^2^ g^–1^)	average pore diameter (nm)	volume of pores between 1.7 and 300 nm (cm^3^ g^–1^)
		ICP-AES	XRD	TEM	XRD	BET	BJH adsorption	
SrFeO_3_	fresh					1.18	29	0.009
	spent					0.92	100	0.003
Ag/SrFeO_3_	fresh	15.7	15.7	124 ± 44	33 ± 12	0.92	59	0.013
	spent	12.5	19.3	86 ± 32	26 ± 3	3.59	85	0.008
Cu/SrFeO_3_	fresh	10.4	13^b^	71 ± 20	25 ± 5^b^	2.87	166	0.010
	spent	6.7^c^	15	234 ± 71	29 ± 4	4.35	85	0.013

a Reported errors represent a single standard deviation from the mean.

b Calculated based on CuO.

c Based on two measurements.

**Figure 4:**
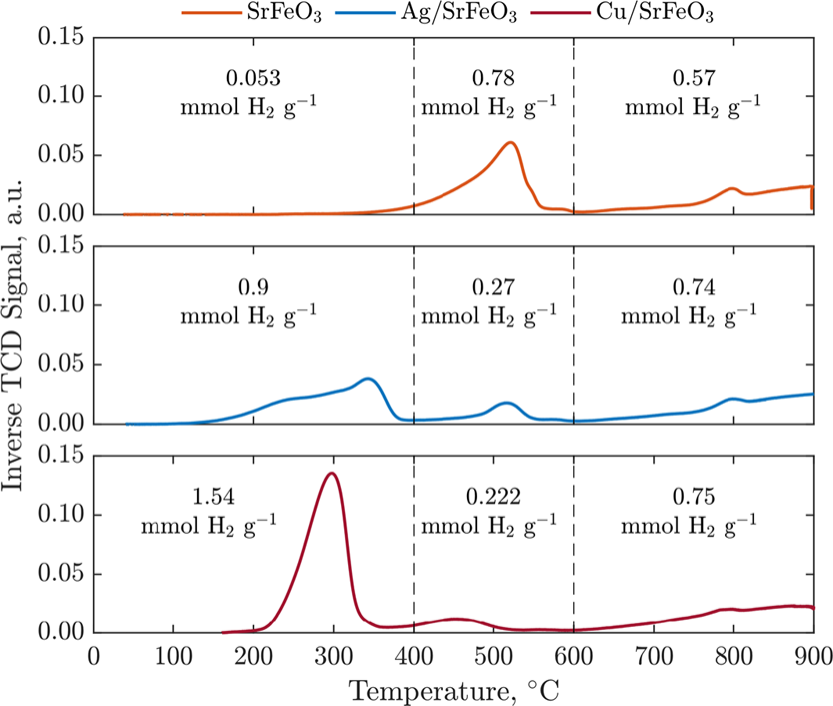
Results from H_2_-TPR experiments performed in 5 vol % H_2_ from 50 to 900 °C with a temperature ramp rate of 10 °C min^–1^. The samples were cleaned in air at 650 °C for 5 h prior to the H_2_-TPR experiment. The H_2_ consumption is reported as mmol H_2_ consumed per gram of SrFeO_3_.

**Figure 5:**
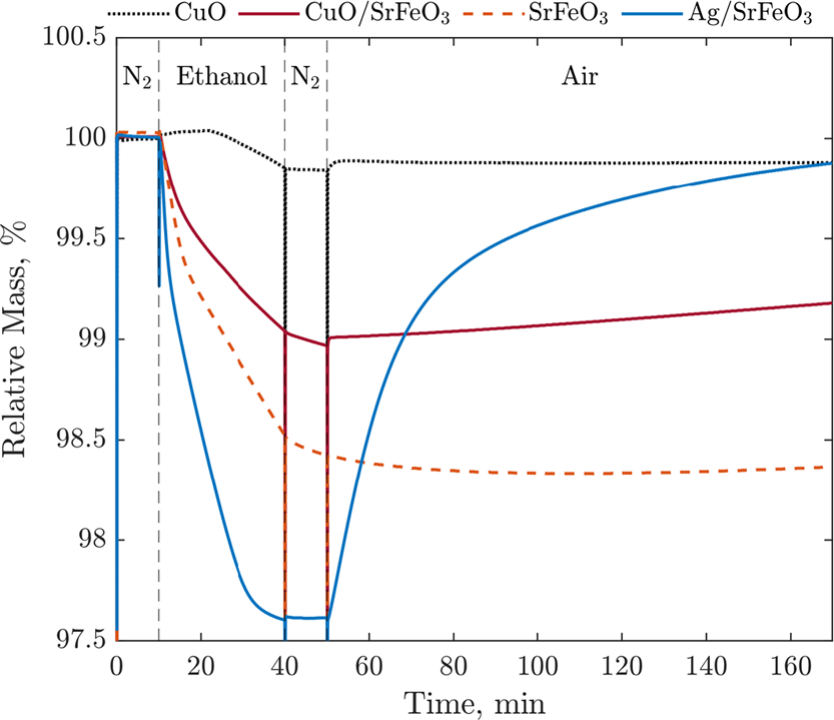
Isothermal TGA experiments conducted at 250 °C with 50 mL min^–1^ “reactive” gas flow of N_2_ for 10 min, ∼3.9 vol % ethanol for 30 min, N_2_ for 10 min, and blended air for 2 h. Fresh samples of Ag/SrFeO_3_, SrFeO_3_, CuO/SrFeO_3_, and CuO on mayenite were used.

Results from the H_2_-TPR experiments shown in Figure 4 clearly demonstrate that adding Ag or Cu to SrFeO_3_ enhances oxygen release in the low-temperature region, that is, up to 400 °C. For the CuO/SrFeO_3_ and Ag/SrFeO_3_ samples, the most pronounced oxygen loss happened between 180 and 380 °C, in the temperature range used during the CL experiments, while the bare SrFeO_3_ reacted much slower and only above 350 °C. Compared to the oxygen release from SrFeO_3_ from the start of the TPR to 400 °C, the addition of Ag and Cu resulted in 0.85 and 1.51 mmol higher consumption of H_2_ per gram of SrFeO_3_, respectively. The majority of the H_2_ consumed from 180 to 350 °C over CuO/SrFeO_3_ took part in the reduction of CuO to Cu^0^ (1.39 mmol H_2_), in addition to the reduction of SrFeO_3_ (0.15 mmol H_2_). However, after the oxygen from CuO depletes, the following regeneration is unsuccessful, as seen from the isothermal CL-cycles in the TGA in Figure 5. Looking at the total H_2_ consumption, that is, up to 900 °C, the samples of SrFeO_3_, Ag/SrFeO_3_, and CuO/SrFeO_3_ consumed 1.4, 1.9, and 2.5 mmol H_2_ g^–1^ SrFeO_3_, respectively; thus, accounting for CuO reduction, SrFeO_3_ released the least amount of oxygen in the last sample, while the presence of silver resulted in enhanced oxygen release.

The re-oxidation of CuO, CuO/SrFeO_3_, SrFeO_3_, and Ag/SrFeO_3_ following reduction in ethanol was assessed through a TGA experiment with isothermal gas switching, as shown in Figure 5. The sample of bare SrFeO_3_ was more deeply reduced than CuO/SrFeO_3_, further demonstrating that CuO hinders the reduction of SrFeO_3_ in ethanol, with CuO itself reducing slowly. The samples containing Cu did not fully re-oxidize, nor did bare SrFeO_3_, all exhibiting slow rates of reoxidation at 250 °C. The presence of Ag enhanced oxygen release and uptake from SrFeO_3_ and resulted in the sample reducing to the largest extent among all samples but also almost completely re-oxidizing at 250 °C, reaching 98.9% of the starting mass after 15 min and 99.5% after 45 min.

**Figure 6:**
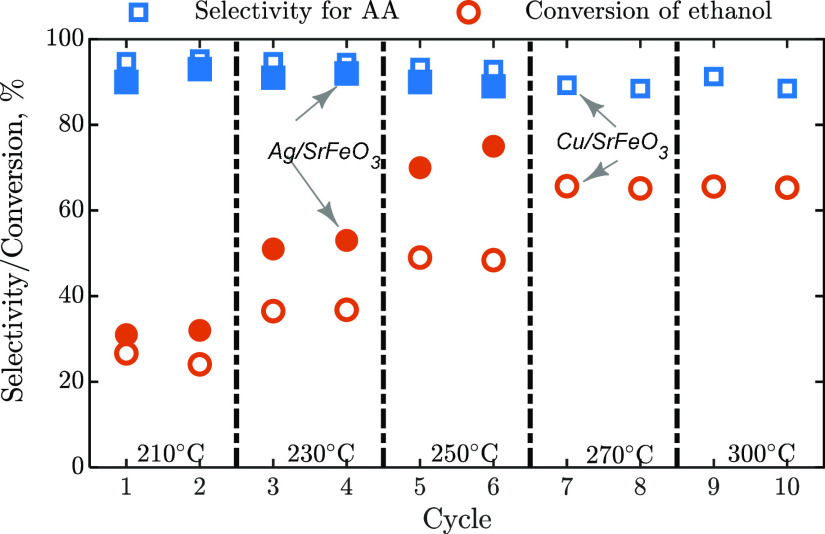
Selectivity for AA and conversion of ethanol during 1.5 min reduction in chemical looping experiments (packed bed) for Ag/SrFeO_3_ (full symbols) and Cu/SrFeO_3_ (empty symbols).

For the two most active and selective samples for AA, the CL experiments were also carried out, mapping the performance indicators with temperature; results are presented in Figure 6. Both samples, Ag/SrFeO_3_ and Cu/SrFeO_3_, showed similar selectivity to AA, but the sample with Ag gave significantly higher conversion of ethanol. Here, experiments with Ag/SrFeO_3_ were noticeably exothermic, with the noticed increase in the bed temperature of up to 20 °C during the 1.5 min reduction steps at 250 °C. Because of the possibility of a runaway reaction, the cycles with Ag/SrFeO_3_ were not carried out above 250 °C. The observed exothermic character is expected from the ODH reaction (R2), with energy being released from combusting hydrogen. In contrast, no significant temperature change was observed in experiments with Cu/SrFeO_3_, which suggests a non-oxidative reaction pathway at play. Even when accounting for the rise in the temperature during the experiments with Ag/SrFeO_3_, the sample with Ag was significantly more active than Cu/SrFeO_3_, producing noticeably more AA per cycle.

**Figure 7:**
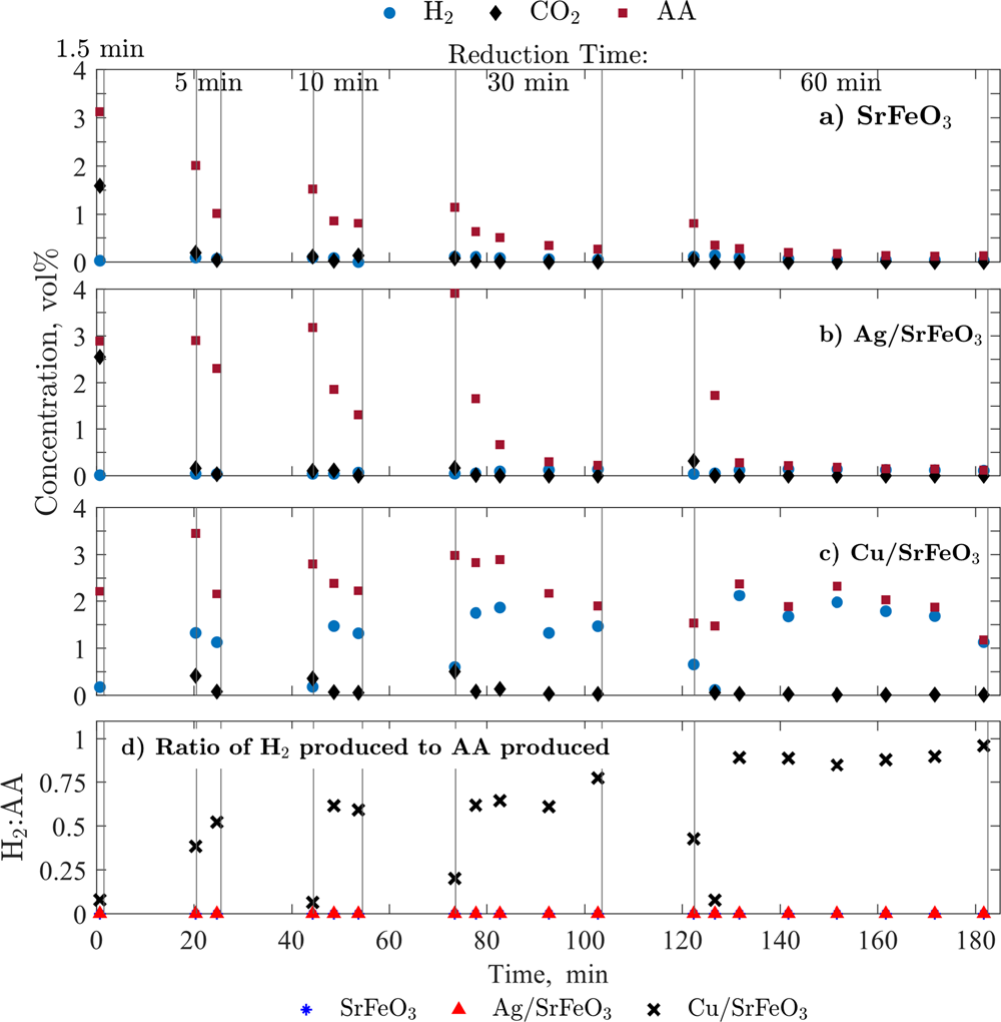
Concentrations of AA, CO_2_, H_2_, and ethanol measured with GC during experiments over (a) SrFeO_3_, (b) Ag/SrFeO_3_, and (c) Cu/SrFeO_3_ with varied times of reduction (1.5 to 60 min) and 15 min oxidation; (d) ratio of H_2_ and AA produced by the three materials; the H_2_:AA ratio for SrFeO_3_ and Ag/SrFeO_3_ is 0 and the symbols fully overlap.

To investigate which reaction model dominated during the CL dehydrogenation of ethanol, the packed bed experiments were repeated, but this time the reduction step was varied from 1.5 to 60 min, with sampling the outlet gas for GC analysis GC at 0.67, 5, 10, 20, 30, 40, 50, and 60 min, or until the reduction step ended. The outlet gas was also analyzed with FTIR, and the measurements from both analyzers agreed. Each reduction step was followed by 15 min reoxidation. The results are presented in Figure 7, giving the ratio of H_2_ to AA produced, as shown in Figure 7d. The H_2_ to AA ratio gives an indication of the extent to which the process behaved non-oxidatively, expecting a 1:1 molar ratio of H_2_ to AA in an ideal non-oxidative dehydrogenation process (R1).

The lack of H_2_ and low levels of CO_2_ in experiments with SrFeO_3_ and Ag/SrFeO_3_ confirmed the proposed CL-ODH reaction pathway, with H_2_ oxidized to H_2_O simultaneously with dehydrogenation of ethanol. The GC setup did not allow for H_2_O detection; however, the FTIR detected that H_2_O accompanied the production of AA at roughly the stoichiometric proportions of R2 over SrFeO_3_ and Ag/SrFeO_3_ (FTIR results are shown in Figures S4 and S5). Since no combustion of ethanol was observed, the combustion of H_2_ on the surface of the perovskite, with or without Ag, was selective. A closer look at the GC results revealed that other organic components (ethylene, diethyl ether, and ethyl acetate) were detected but only at low-ppm levels, further corroborating the dominating nature of the CL-ODH pathway. Additionally, both SrFeO_3_ and Ag/SrFeO_3_ became inactive after 10 min of exposure to ethanol. Given the lack of CO_2_ released during reoxidation, the deactivation at 10 min is likely a result of the materials becoming depleted of oxygen, demonstrating the dependence on oxygen and confirming the CL-ODH reaction. From the group’s previous work,^26^ below 300 °C, Ag/SrFeO_3_ donates more oxygen than SrFeO_3_, explaining the difference in the CL activity seen in Figure 7a,b.

**Figure 8:**
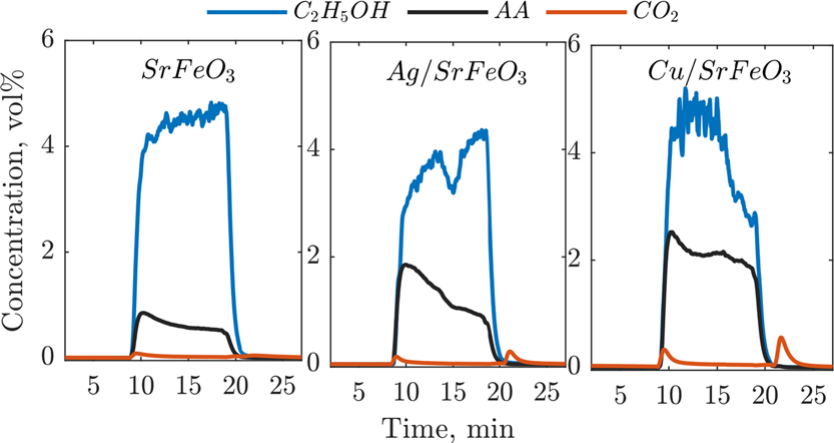
Measured concentrations of AA, CO_2_, and ethanol measured with FTIR during a 10-min reduction step. Experiments with SrFeO_3_ and Ag/SrFeO_3_ were carried out with 5.4 vol % of ethanol, while the experiment with Cu/SrFeO_3_ was initially carried out with 6.4 vol % of ethanol because of problems with ethanol feeding.

**Figure 9:**
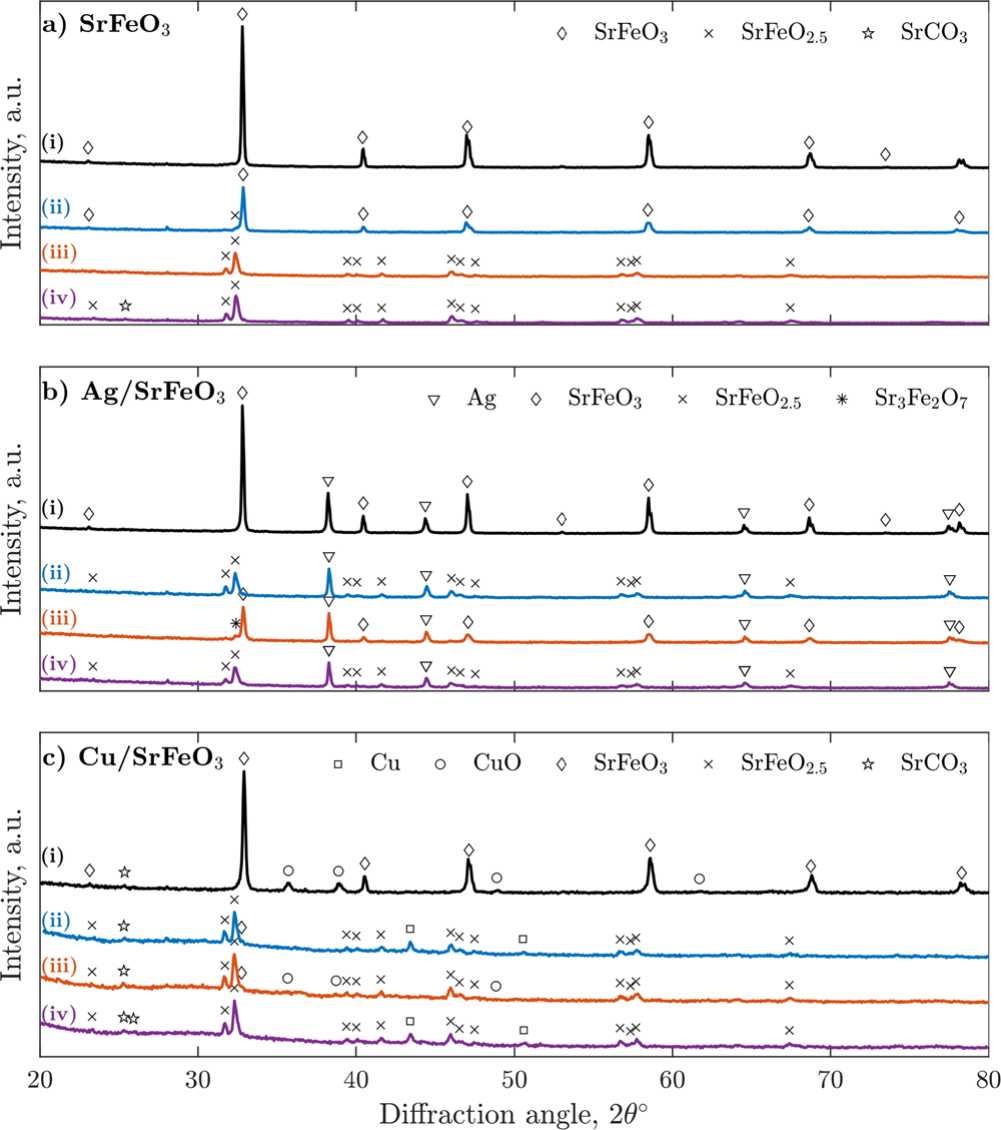
Recorded XRD patterns of (a) SrFeO_3_, (b) Ag/SrFeO_3_, and (c) Cu/SrFeO_3_ as (i) fresh material, (ii) after 1.5 min of reduction in ethanol without reoxidation, (iii) after 10 cycles of 1.5 min reduction in ethanol and 15 min regeneration in air, and (iv) after 60 min of reduction in ethanol without reoxidation. The experiments ii–iv were conducted sequentially for each material, with the material being cooled in N_2_ and sampled between experiments.

In contrast to SrFeO_3_ and Ag/SrFeO_3_, which supported AA production for ∼10 min, the catalyst containing Cu/SrFeO_3_ was selective toward AA for up to 60 min of the continuous reduction step (Figure 7c). Noticeable concentrations of CO_2_ and low concentrations of H_2_ at the start of each cycle confirm that oxidized forms of Cu (Cu_2_O and CuO) combusted ethanol unselectively. Once Cu was created (confirmed with XRD, Figure 9c-ii), the metallic Cu started to catalyze non-oxidative dehydrogenation of ethanol, accompanied by H_2_ production. A portion of the cogenerated H_2_ was at first combusted on SrFeO_3_, with H_2_O detected with FTIR for significantly longer than CO_2_ (results in Figures S6 and S7). As shown in Figure S8, the Cu/SrFeO_3_ catalyst had a large portion of the perovskite support exposed; thus, SrFeO_3_ could reduce regardless of reactions involving Cu. The presence of SrFeO_3_ prevented severe coking (or deposition of organic products); after 10 min reduction (Figure 8) and with oxygen from SrFeO_3_ also used for H_2_ combustion, the amount of CO_2_ during coke removal was similar to that observed after 1.5 min reduction (Figure 2).

Continuous FTIR measurements of the composition at the reactor outlet during the experiment with 10 min reduction are presented in Figure 8. Noticeably, the production of AA with SrFeO_3_ and Ag/SrFeO_3_ was sustained through the whole reduction step, demonstrating that CL-ODH can be carried out for longer than 1.5 min used in the first set of experiments (Figure 2).

Results from the XRD analysis of fresh and spent samples are shown in Figure 9, with a more detailed overview also in Figures S9–S11. As intended, only CuO, Ag, and SrFe perovskites were detected in the fresh CL samples. During experiments with Ag/SrFeO_3_, the silver remained as metallic silver, while in CuO/SrFeO_3_, the CuO easily reduced to metallic copper, with only minimal quantities of CuO regenerated in 15 min reoxidation. Following the 1.5 min reduction in ethanol, the amount of the cubic perovskite (SrFeO_3_) decreased in all samples, accompanied by an increase in the orthorhombic and oxygen-reduced brownmillerite phase (SrFeO_2.5_). For the two most active samples, Ag/SrFeO_3_ and CuO/SrFeO_3_, only brownmillerite was detected, confirming the larger extent of oxygen depletion. All spent materials were sampled from the top of the bed; thus, the XRD results represent only the most reduced particles rather than the average of the experimental samples. A picture of the bed after experiments in Figure S12 reveals that only part of the bed reduced to SrFeO_2.5_.

The XRD results indicated that SrCO_3_ was present in similar amounts in the fresh (∼6 wt %) and spent (∼8 wt %) Cu samples, indicating only a little effect of the reduction in ethanol. The XRD patterns for Ag/SrFeO_3_ samples did not have any SrCO_3_ peaks present above noise, and SrCO_3_ was only observed in the bare SrFeO_3_ sample after 60 min of reduction in ethanol.

**Figure 10:**
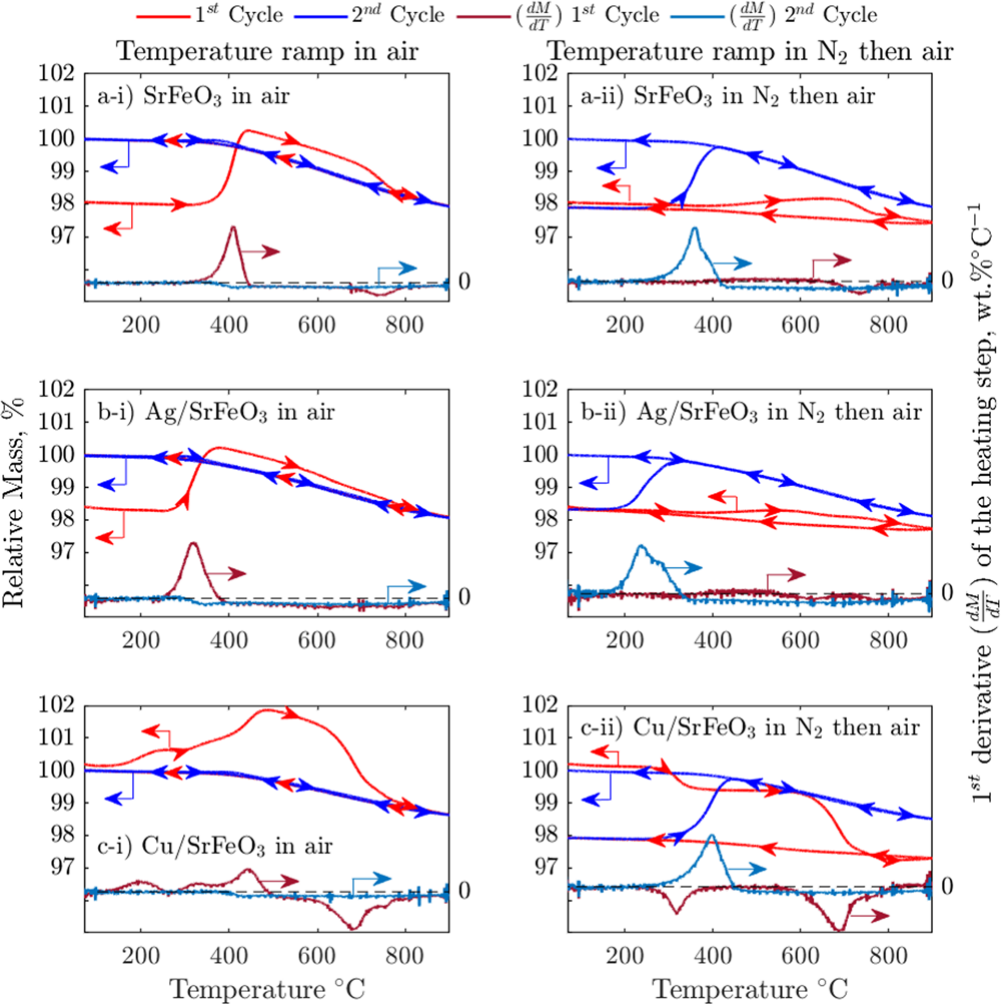
Relative mass and *dm*/*dt* recorded during the TGA experiments with spent (a) SrFeO_3_, (b) Ag/SrFeO_3_, and (c) Cu/SrFeO_3_. The TGA experiments comprised temperature programmed heating and cooling between 50 and 900 °C in (i) air or (ii) N_2_ followed by air. The derivatives, *dm*/*dT*, are shown only for the heating steps.

The spent samples of SrFeO_3_, Ag/SrFeO_3_, and Cu/SrFeO_3_ were further analyzed in TGA, using temperature cycles, ramping from 50 to 900 °C, under a flow of “reactive” gases: (i) air, (ii) CO_2_, and (iii) N_2_, with the results shown in Figure 10. The heating step in air in the second TGA cycle represents the typical mass change expected from a catalyst, while the same step in the first cycle represents additional mass changes connected to oxygen uptake by SrFeO_3_ and decomposition/surface cleaning reactions. Comparison of those two heating steps reveals the extent of reduction of SrFeO_3_ and the accumulation of surface impurities, coke, and/or carbonates, caused by the 60 min exposure to ethanol in the packed bed reactor. To help with the analysis, first derivatives of mass loss during the heating steps are also shown in Figure 10. During heating to ∼450 °C in air, each of the spent samples gained mass, resulting from the incorporation of oxygen into the SrFeO_3_ lattice and oxidation of Cu^0^ in the case of Cu/SrFeO_3_. Upon further heating to 900 °C, SrFeO_3_ released oxygen, as observed with a mass drop. The mass loss in the first cycle was greater than in the second, the difference explained by the removal of carbon species at the surface of each sample, with the lowest difference between cycles for Ag/SrFeO_3_, indicating the smallest amount of removable coking or carbonates, in agreement with the XRD results shown in Figure 9. Notably, CuO is stable over the investigated temperature range for the applied oxygen partial pressure (0.07 bar), as shown in Figure S13, so CuO did not contribute to the observed mass loss.

From Figure S14, in TGA, SrCO_3_ begins to decompose in air at ∼700 °C; however, the presence of SrFeO_3_, Ag, and Cu might affect the decomposition of carbonates;^29^ for example, SrCO_3_ has been shown to decompose on Ce-doped SrFeO_3_ already at 610 °C.^30^ The gradient of mass loss over bare SrFeO_3_ in the first cycle differed to that in the second cycle at temperatures between 670 and 800 °C, with a 0.57 wt % greater mass loss observed in the first cycle. A similar mass loss of 0.64 wt % occurred over 690–900 °C when the experiment was conducted in N_2_, which strongly points toward the presence of SrCO_3_ rather than coke. Expectedly, coke removal would occur only in experiments in air because coke removal requires oxygen or a gasifying species (H_2_O, CO_2_); the same mass loss in experiments in air and N_2_ indicates SrCO_3_ as the main surface impurity.

Considering Ag/SrFeO_3_, the mass loss in the first cycle in air deviates from that in the second cycle, with the difference in derivatives revealing two distinct periods of greater mass loss, between 480–660 and 710–823 °C, observed in the first cycle. The additional mass loss of 0.17 wt % over 710–823 °C can be again attributed to the SrCO_3_ removal; however, the mass loss of 0.28 wt % over 480–660 °C is unlikely for SrCO_3_ decomposition because of the low temperatures; thus, it must have resulted from the coke removal.

The TGA results for the Cu/SrFeO_3_ sample displayed no distinct regions that can be ascribed to coke or SrCO_3_ removal in air, with the mass loss in the first cycle being consistently greater than the mass loss in the second cycle. The spent Cu/SrFeO_3_ contained the most removable impurities, as the difference in the total mass loss in the first and second cycle in air was 2 wt % greater than those for Ag/SrFeO_3_ or SrFeO_3_. The presence of coke on the spent samples was assessed with Raman spectroscopy, with results presented in Figure S17. While clear features were observed for SrFeO_3_ and deposited metallic particles, for coke, only a very weak peak at 1580 cm^–1^ was detected.^31^ The weak signal from the coke might be partially explained by high-intensity features from SrFeO_3_ to which the signal was normalized, leaving the small intensity features of coke or carbonates as barely protruding from the background.

Overall, both SrFeO_3_ and Ag/SrFeO_3_ contained very low levels of surface impurities, despite Ag/SrFeO_3_ being roughly twice as active as SrFeO_3_. While Cu/SrFeO_3_ accumulated the largest amount of impurities when exposed to ethanol, it was also active for the longest time, as seen in Figure 7. Further results from the TGA, with cycles performed in CO_2_ followed by two cycles in air, are shown in Figure S15; clearly, the Cu/SrFeO_3_ sample contained the most carbonates following the exposure to CO_2_. Thus, among the investigated samples, Cu/SrFeO_3_ appears to be the most prone to carbonation (also confirmed by the XRD results in Figure 9), while still remaining active in the long experiments (Figure 7).

The Cu/SrFeO_3_ material clearly facilitated a non-oxidative dehydrogenation route (R1), evidenced by the co-production of AA and H_2_. However, the production of water was also observed over Cu/SrFeO_3_ due to the oxidation of H_2_, expected in the oxidative dehydrogenation route (R2). Thus, both oxidative and non-oxidative dehydrogenation occurred simultaneously over Cu/SrFeO_3_. The oxygen required to oxidize H_2_ was provided from the perovskite support, as the XRD analysis (Figure 9) following 1.5 and 60 min reduction revealed a severely reduced perovskite phase and Cu to be the only copper phase. Additionally, Cu mapping of the Cu/SrFeO_3_ material with EDS, shown in Figure S8, found that large portions of the SrFeO_3_ support remain exposed following the copper impregnation method. Thus, the exposed SrFeO_3_ facilitated the oxidative dehydrogenation method, and the Cu supported the non-oxidative pathway. However, to what extent the H_2_ produced on Cu migrated and oxidized over SrFeO_3_ could not be determined.

**Figure 11:**
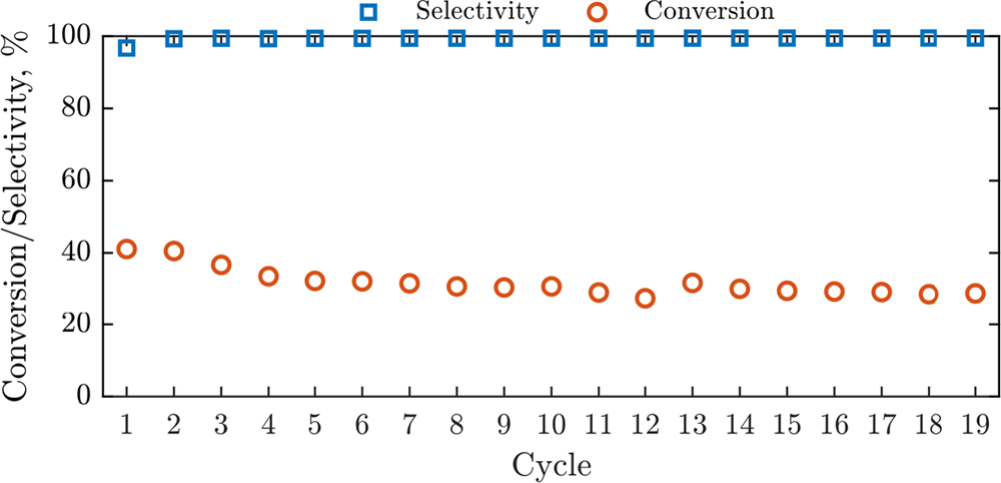
Performance during 19 CL-ODH cycles with 5 min reduction and 15 min oxidation over 2 g Ag/SrFeO_3_.

Since the production of AA on Ag/SrFeO_3_ was most pronounced in the first 5 min of exposing the catalyst to ethanol, the next set of experiments was conducted with a reduction step of 5 min, looking at the performance in multiple CL-ODH cycles. Results are presented in Figure 11. The obtained selectivity to AA reached almost 100%, with the conversion of ethanol >30%. This performance can be extended into a pseudo-continuous production of AA when CL reactors are run in parallel, requiring 4 CL reactors operating in 5 min reduction and 15 min oxidation steps. Results in Figures 7 and 8 indicate that the reduction step could be extended further, as AA is still produced after 10 min of exposure to ethanol. Therefore, using the experimental setup with 2 g of Ag/SrFeO_3_ and 200 mL/min flowrate of ∼5.8 vol % C_2_H_5_OH, a pseudo-continuous production of AA could be achieved with 3 CL-ODH reactors only.

**Figure 12:**
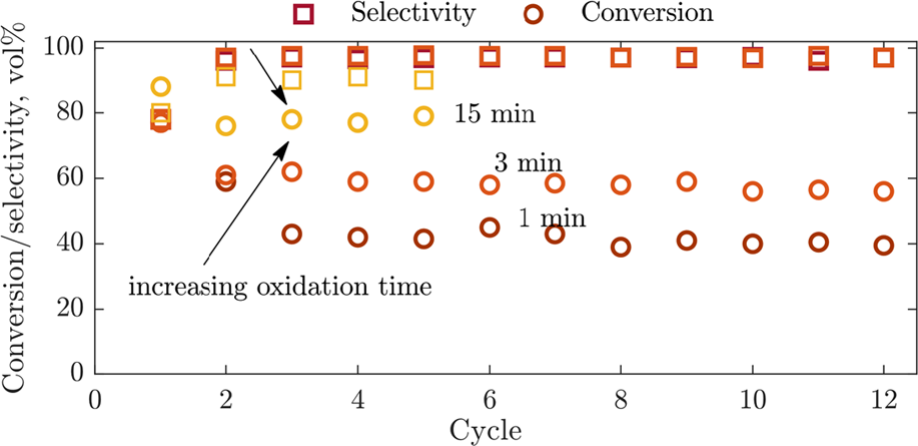
Performance during CL-ODH over 2 g Ag/SrFeO_3_ cycles with 1.5 min reduction and varied oxidation times from 1 to 15 min.

The opportunity to optimize the process is further demonstrated in Figure 12, this time looking at the oxidation time. Decreasing the oxidation time from 15 min to 3 or 1 min improved the selectivity to AA at the cost of lower conversion of ethanol. The oxygen chemical potential (μ_O_) of the catalytic material factors into the selectivity of oxidation reactions, with high μ_O_ potentially leading to excessive oxidation, while low μ_O_ resulting in inactivity.^32^ The chemical potential μ_O_ of the SrFeO_3_ materials decreased during the reduction step, but during the regeneration step in air, as oxygen was re-incorporated into the lattice, μ_O_ increased until the regeneration step ended, or the sample was fully re-oxidized. Clearly, the prolonged oxidation of Ag/SrFeO_3_ leads to the consequent overoxidation of a fraction of the products (lower selectivity to AA), but with the benefit of more ethanol participating in the CL reaction. Very good performance can be achieved with 3 min oxidation and 1.5 min reduction, equivalent to three CL-ODH reactors working in parallel for a pseudo-continuous CL-ODH process.

In this work, experiments focused on a packed-bed configuration, primarily to avoid attrition of the Ag catalyst from the surface of SrFeO_3_ particles. Since the oxygen carrier is not utilized uniformly in the bed (see Figure S12), other reactor designs can be considered. Recently, some new configurations for CL processes have been recently proposed,^33−35^ indicating further opportunities.

## 4 Conclusions

AA was produced by passing ethanol through a bed of CL materials capable of donating oxygen. The best performance was achieved with Ag/SrFeO_3_, with AA being produced via oxidative dehydrogenation, where oxygen donated by SrFeO_3_ combusted H_2_. When Ag was replaced by Cu, the reaction pathway switched to a mixed oxidative and non-oxidative dehydrogenation, with the SrFeO_3_ supporting the oxidative reaction pathway and Cu the non-oxidative reaction. A better performance was achieved with Ag/SrFeO_3_ as the CL material in the CL-ODH cycles than with Cu/SrFeO_3_ in the mixed oxidative and non-oxidative reactions. The CL-ODH cycles can be adjusted to minimize the oxidation time or extend the reduction time, optimizing for the highest AA yields and minimizing the number of CL-ODH reactors running in parallel.

## Supporting Information Available

Results from CL experiments using GC, size distribution of Cu and Ag particles measured from STEM images, results from CL experiments with varied reduction times, SEM-EDS image of a fresh CuO/SrFeO_3_ sample, XRD results of fresh and spent SrFeO_3_-based materials, picture of a reduced bed of SrFeO_3_, phase diagram for CuO, TGA experiments with SrCO_3_ and SrFeO_3_-based materials, and Raman analysis of spent and fresh samples of SrFeO_3_-based materials (PDF)
