# Supplementary material for: Production of Acetaldehyde via Oxidative Dehydrogenation of Ethanol in a Chemical Looping Setup
Source: ACS Eng Au. 2023 Feb 28;3(3):184–94. doi: 10.1021/acsengineeringau.2c00052 (PMC10288508; doi:10.1021/acsengineeringau.2c00052)
Supplement: Supplementary file 1 — eg2c00052_si_001.pdf [file eg2c00052_si_001.pdf]

Supplementary Information

Production of acetaldehyde *via* the oxidative dehydrogenation of ethanol in a chemical looping setup

Joseph C. Gebers, Abu Farhan Bin Abu Kasim, George J. Fulham, Kien Yi Kwong, Ewa J. Marek*

Department of Chemical Engineering and Biotechnology, University of Cambridge, Philippa Fawcett Drive, CB3 0AS, United Kingdom

**Corresponding author, ejm94@cam.ac.uk*

1. GC analysis of the gas products from the reduction step

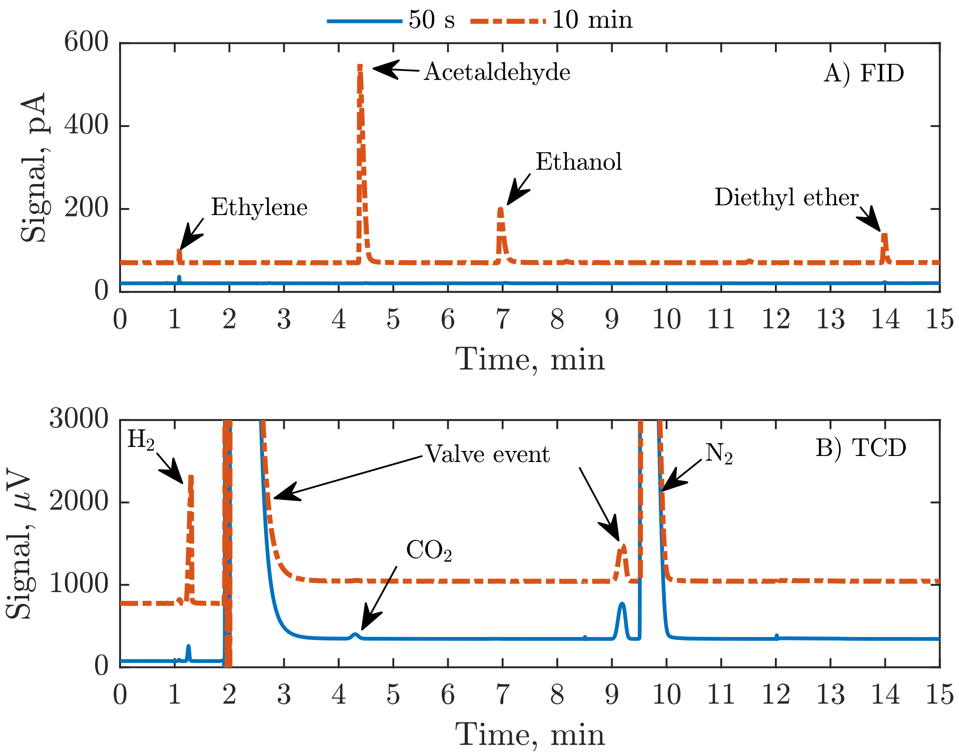

Figure S1: GC results with (A) FID and (B) TCD signals measured at 50 s and 10 min into a reduction step performed with CuO/Al_2O_3 at 250°C. The TCD signals recorded at the retention times of 2 to 3 min, 9 min, and 9.5 min were caused by valve switching events programmed into the GC.

An extended reduction step was performed over CuO/Al_2O_3. Samples of the outlet gas were collected manually at 50 s and 10 min into the reduction step and analysed with the GC; results are shown in Fig. S1**.** Consistent with the CuO/Al_2O_3 chemical looping (CL) profiles shown in Fig. 2 of the main manuscript, no acetaldehyde (AA) (4.4 min) or ethanol (6.8 min) was observed in the FID signal for a sample taken 50 s into the reduction step. Very small amounts of ethylene (1.1 min) and diethyl ether (14 min) were found instead, indicating that a small fraction of ethanol dehydrated over the α-Al_2O_3 support, which was unexpected given the low operating temperature (250°C) and α-Al_2O_3 being largely inactive for dehydration[1]. At the same time, low concentrations of CO_2 (4.3 min) and H_2 (1.2 min) were detected by the TCD.

The absence of AA when H_2 was detected confirms that ethanol cracking dominated early in the reduction step.

The GC samples taken 10 min into the reduction step yielded considerably more dehydrogenation products. Acetaldehyde was present at 2.1vol%, H_2 at 7.4vol%, whilst unreacted ethanol was also detected, with no CO_2 produced. Again, dehydration products, ethylene and diethyl ether, were also detected, albeit at low concentrations. The prolonged reduction with CuO/Al_2O_3 demonstrates that this Cu-sample becomes selective towards AA only after the first event of prominent coking and carbonation (also see Fig. S10 for the XRD results of the samples after 1.5 or 60 min of reduction).

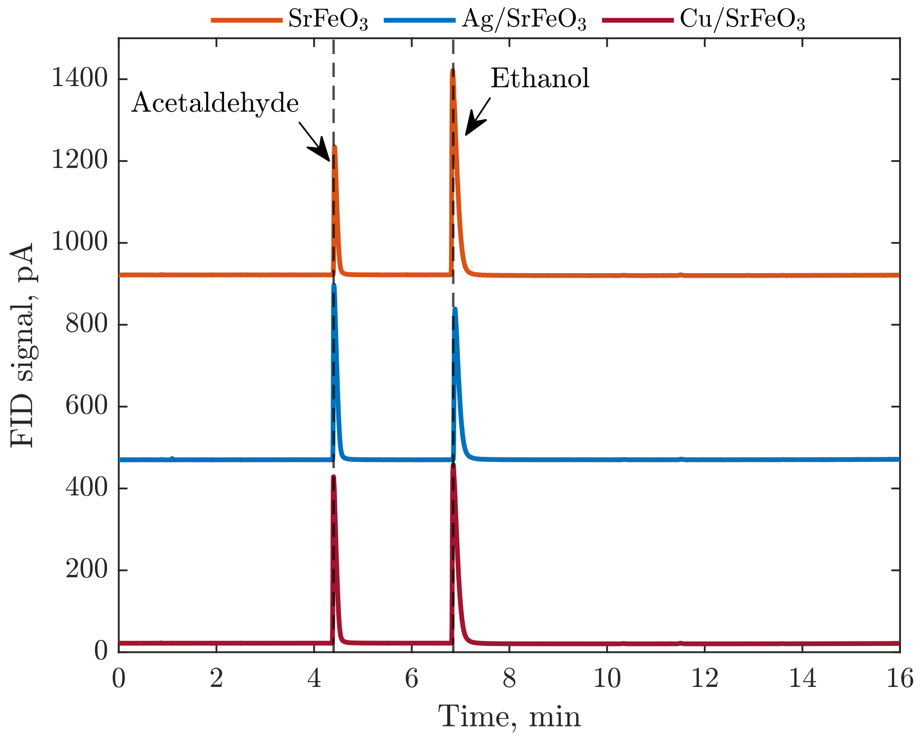

Figure S2: The FID results from GC measurements taken during the reduction step of the 3rd CL cycle over $SrFeO_3$, $Ag/SrFeO_3$, and $Cu/SrFeO_3$.

Typical FID results of GC measurements taken during the reduction step are shown in Fig. S2. Acetaldehyde and ethanol are present at the 2 to 4vol% range. Whilst other hydrocarbons can be detected, none exhibited an FID response greater than 3 pA, which corresponds to concentrations in the 10s and 100s of ppm range.

2. Size distribution of Cu and Ag particles in spent and fresh samples

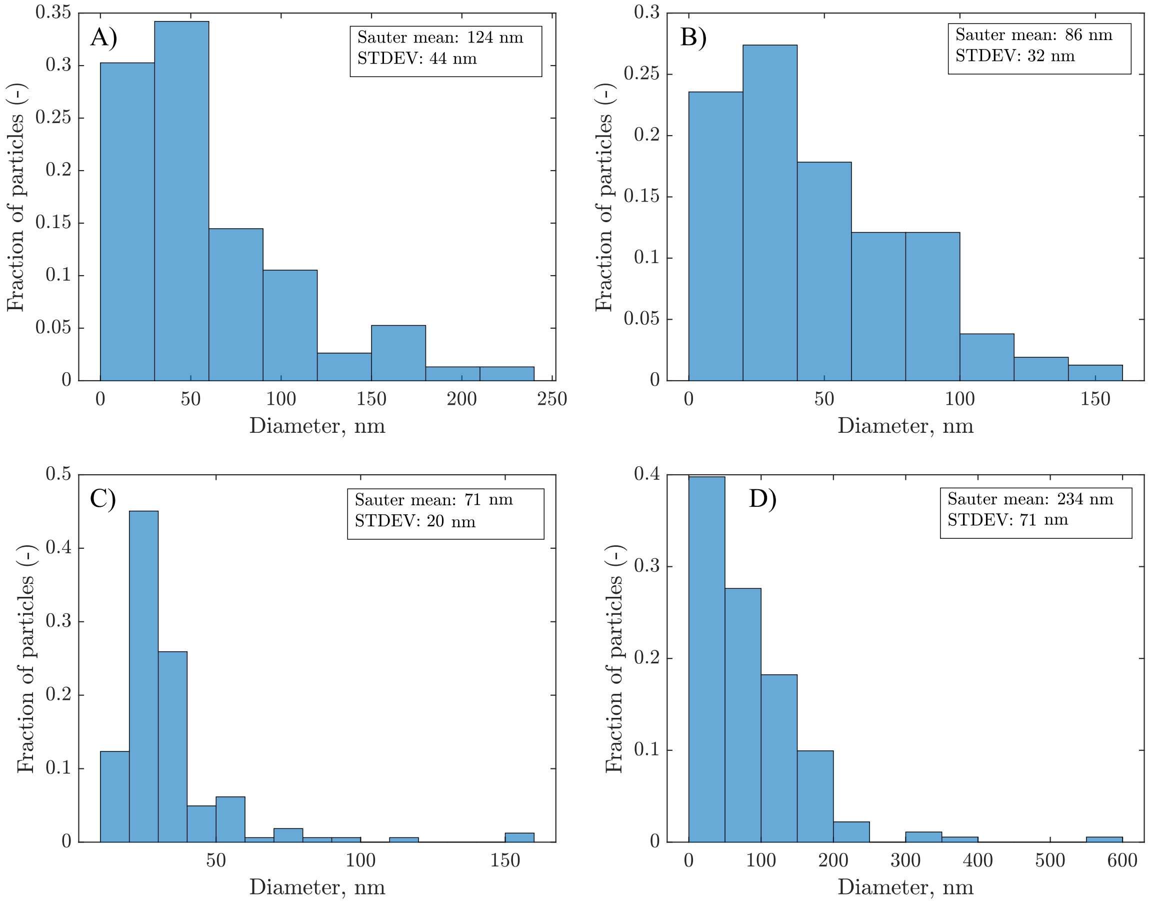

Figure S3: Histograms showing the size distributions of Ag particles in A) fresh and B) spent Ag/$SrFeO_3$, C) CuO particles in fresh CuO/$SrFeO_3$, and D) Cu particles in spent Cu/$SrFeO_3$. The Sauter mean diameter and standard deviation of the particles is reported.

The surface-weighted mean diameter (Sauter diameter), $d_{3,2}$, was calculated by Eq. S1 using particle diameters, d_i, that were manually measured from STEM images and EDS maps of fresh and spent Ag/$SrFeO_3$ and Cu/$SrFeO_3$. At least three different sections of the samples were imaged and no less than 150 particles were measured.

$$d_{3,2} = \frac{\sum d_i^3}{\sum d_i^2} \qquad \text{Eq. S1}$$

3. FTIR measurements of experiments with varied times of reduction over $SrFeO_3$-based materials

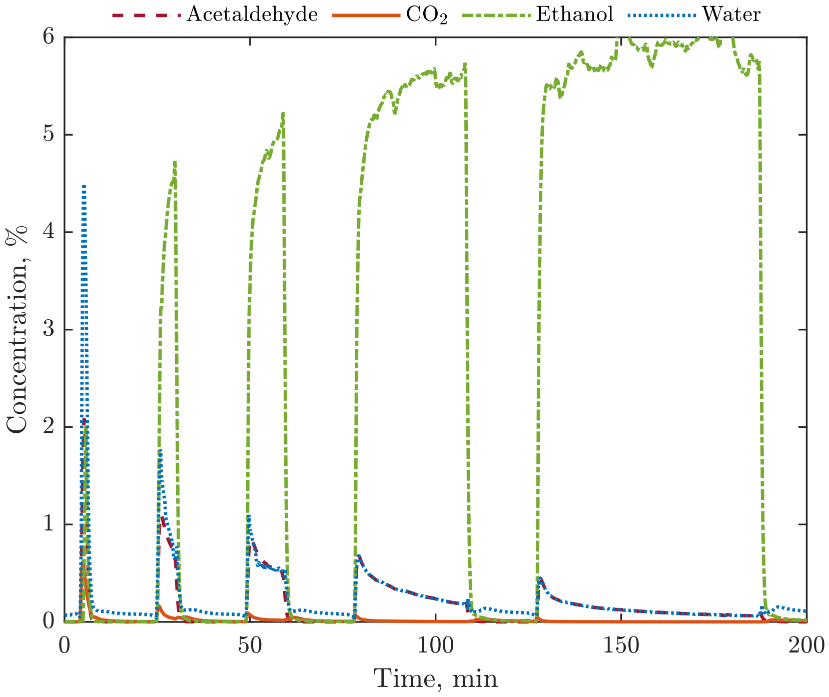

Figure S4: Concentrations of AA, CO_2, H_2O, and ethanol measured with FITR during experiments over $SrFeO_3$, with varied times of reduction (1.5 to 60 min) and 15 min oxidation.

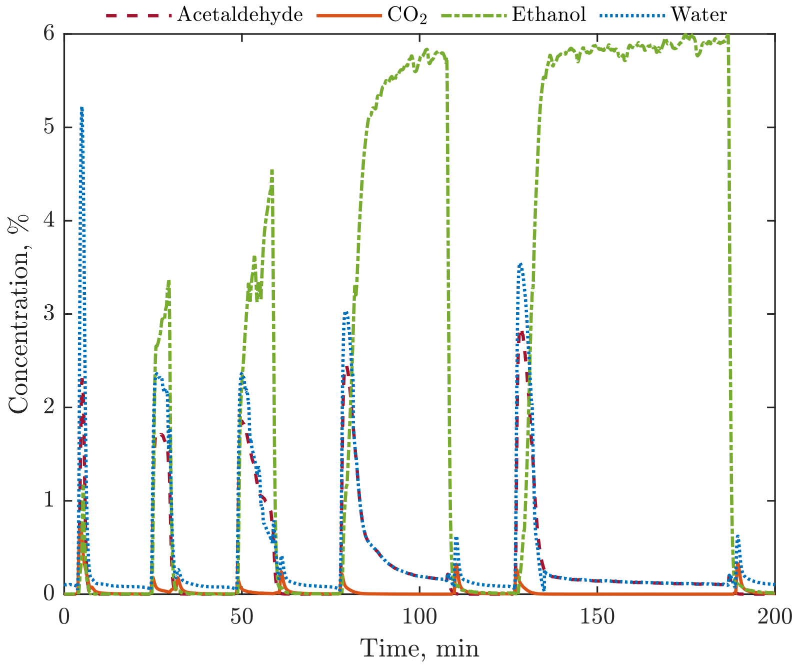

Figure S5: Concentrations of AA, CO_2, H_2O, and ethanol measured with FITR during experiments over Ag/$SrFeO_3$, with varied times of reduction (1.5 to 60 min) and 15 min oxidation.

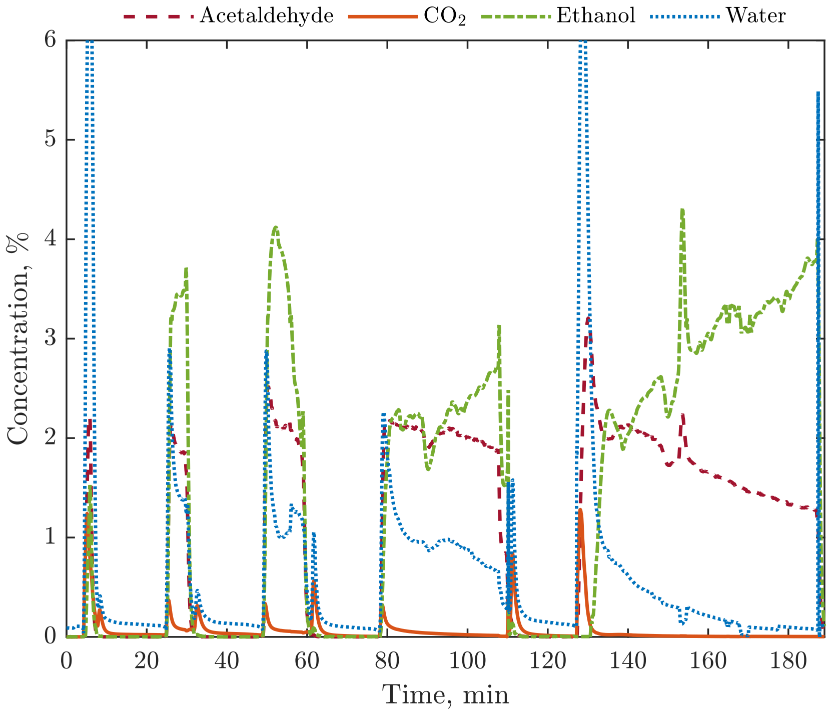

Figure S6: Concentrations of AA, CO_2, H_2O, and ethanol measured with FITR during experiments over Cu/$SrFeO_3$, with varied times of reduction (1.5 to 60 min) and 15 min oxidation.

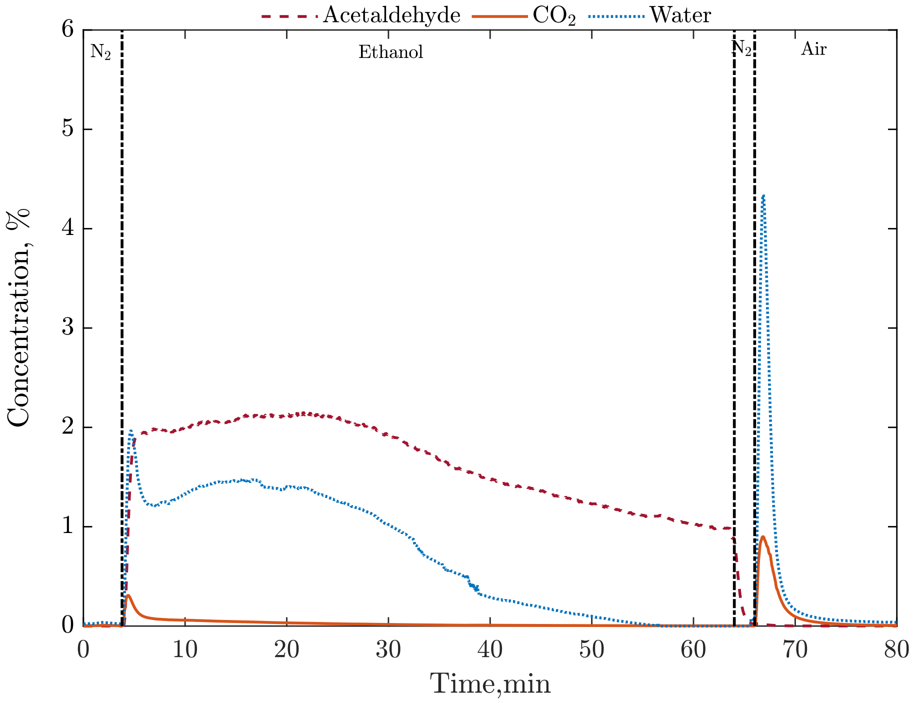

Figure S7: Concentration profiles of acetaldehyde, CO_2, and water during a 60 min reduction of CuO/$SrFeO_3$ in 5.8vol% ethanol, followed by regeneration in air at 250°C.

4. SEM-EDS image of a fresh $CuO/SrFeO_3$ sample

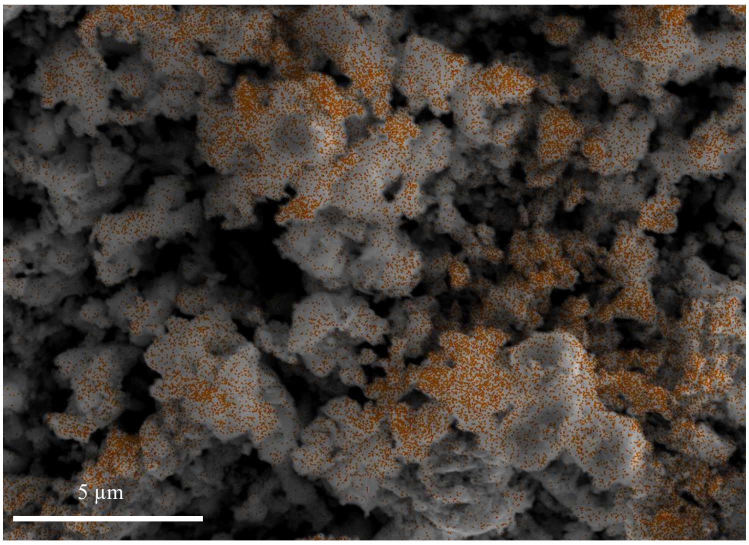

Figure S8: SEM-EDS maps of fresh $CuO/SrFeO_3$ showing the copper distribution (red dots) over $SrFeO_3$.

The surface elemental composition was determined *via* energy-dispersive X-ray spectroscopy (EDS) analysis performed using an Oxford Instruments Aztec Energy X-maxN system with an accelerating voltage of 15 kV and a working distance of 15 mm. A SEM-EDS image of the fresh $CuO/SrFeO_3$ sample is given in Fig. S8.

5. XRD results of spent and fresh $SrFeO_3$-based materials

The following figures (Figs. S9 – S11) present the same XRD results as in Fig. 9 of the main manuscript. Here, plotted over a smaller range of signal intensity to show minor peaks.

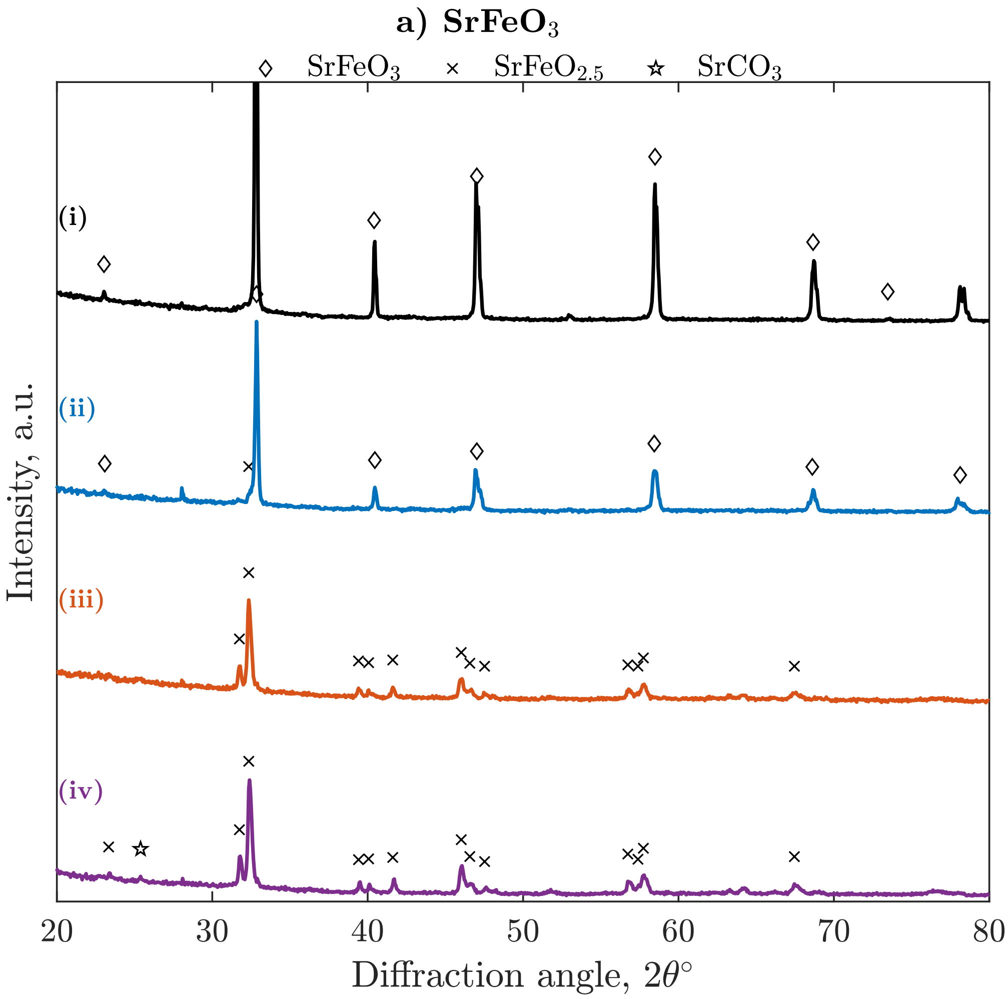

Figure S9: XRD patterns of a) $SrFeO_3$, as i) fresh material, ii) after 1.5 min of reduction in ethanol without reoxidation, iii) after 10 cycles of 1.5 min reduction in ethanol and 15 min regeneration in air, and iv) after 60 min of reduction in ethanol without reoxidation.

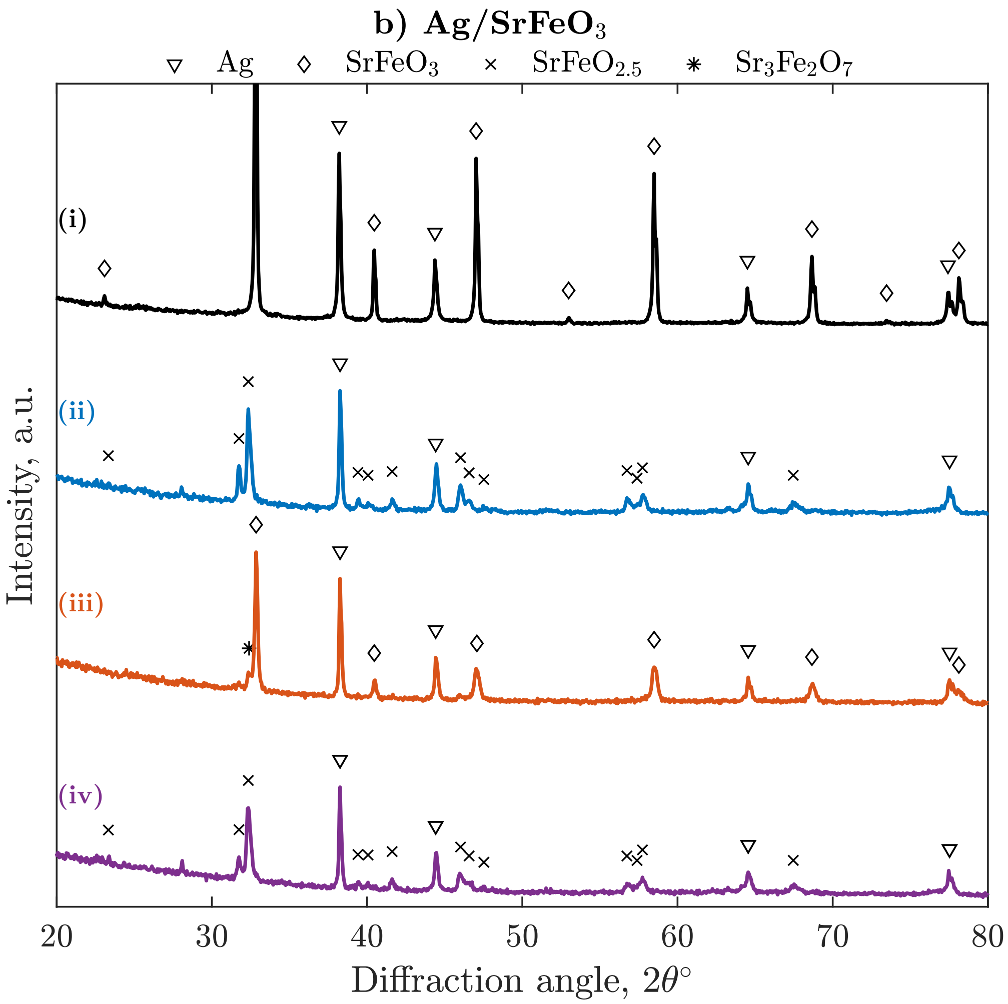

Figure S10: XRD patterns of b) Ag/SrFeO$_3$, as i) fresh material, ii) after 1.5 min of reduction in ethanol without reoxidation, iii) after 10 cycles of 1.5 min reduction in ethanol and 15 min regeneration in air, and iv) after 60 min of reduction in ethanol without reoxidation.

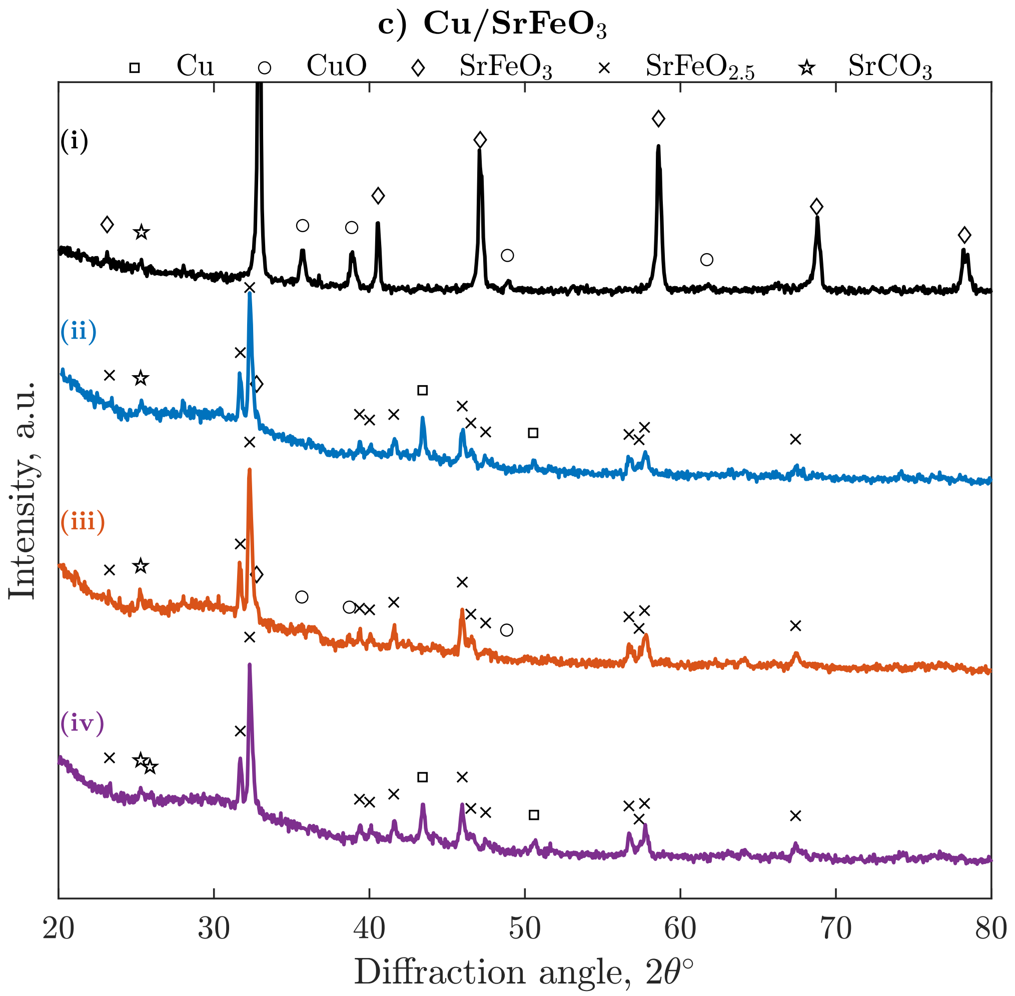

Figure S11: XRD patterns of c) Cu/$SrFeO_3$, as i) fresh material (CuO/$SrFeO_3$), ii) after 1.5 min of reduction in ethanol without reoxidation, iii) after 10 cycles of 1.5 min reduction in ethanol and 15 min regeneration in air, and iv) after 60 min of reduction in ethanol without reoxidation.

6. Reduction of $SrFeO_{3-\delta}$

Inspection of the reactor bed containing $Ag/SrFeO_3$ following 1.5 min of reduction, seen in Fig. S12, showed a clear visible change in the top portion of the bed to $SrFeO_{2.5}$, which corresponds to the section of the bed that was sampled for XRD analysis.

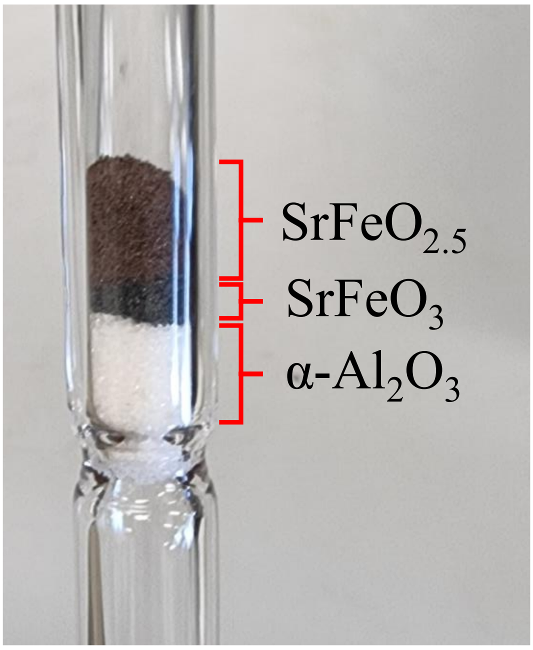

Figure S12: Packed bed of $Ag/SrFeO_3$ catalyst and α-Al_2O_3 inert following 1.5 min of reduction in ethanol at 250°C. The top layer of α-Al_2O_3 was removed to sample particles of the reacted catalyst, thus, is missing in the picture.

7. Copper phases by oxygen partial pressure and temperature

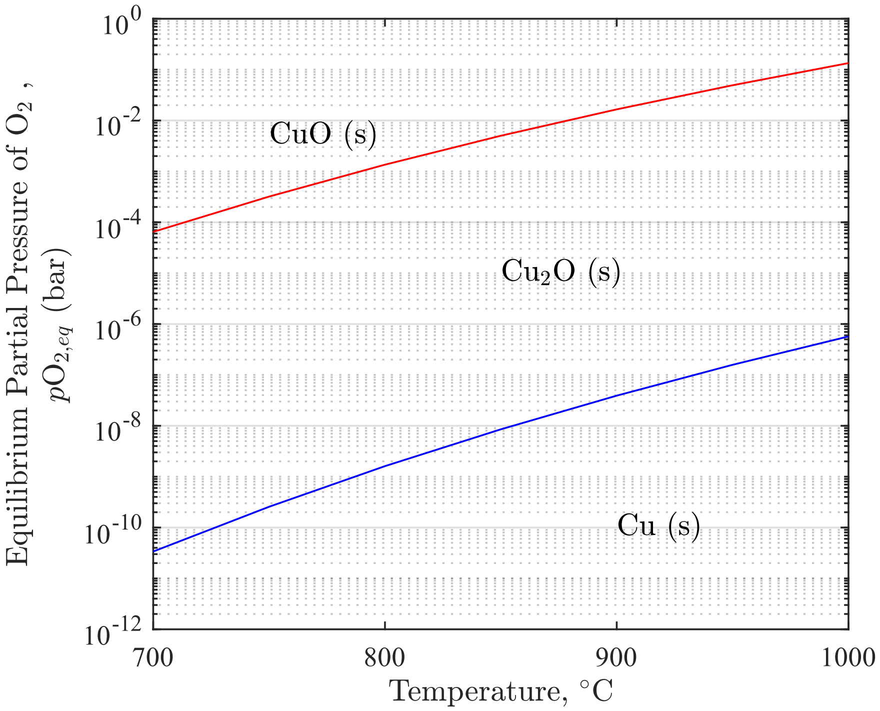

Figure S13: Phase diagram of Cu-O.

8. Additional TGA results of $SrCO_3$ under an air 'reactive' gas and $SrFeO_3$-based materials under a CO_2 'reactive' gas

The decomposition of $SrCO_3$ (Sigma Aldrich, ≥ 98%) over a temperature cycle from 50-900°C with a temperature ramp rate of 10 °C min^{-1} with air as the 'reactive' gas was observed in the TGA, seen in Fig. S14. Under a CO_2-free atmosphere, $SrCO_3$ is expected to be thermodynamically unfavorable, however, the decomposition of $SrCO_3$ to SrO is limited by slow kinetics[3]. The $SrCO_3$ started decomposing at ~700°C and continued to decompose until the TGA had cooled to 800°C.

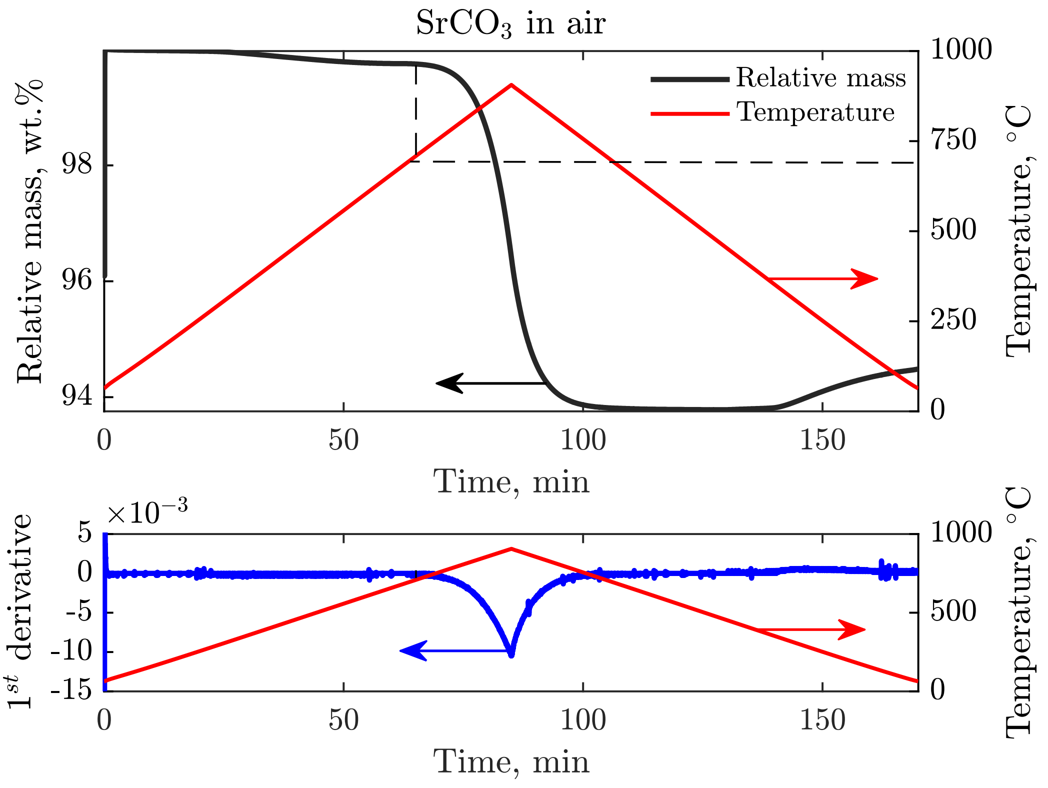

Figure S14: Relative mass change observed during the decomposition of $SrCO_3$ to SrO in a temperature-programmed cycle of 50-900°C with a temperature ramp rate of 10°C min^{-1} under an air 'reactive' gas.

Two temperature cycles were performed in CO_2 with the spent $SrFeO_3$-based materials, shown in Fig. S15, as CO_2 is expected not to re-oxidise samples beyond the $SrFeO_{2.5}$ phase[2], nor remove any $SrCO_3$; whilst coke removal is expected at temperatures above 750°C[4]. Immediately after the cycles in CO_2, two temperature cycles in air were performed, also seen in Fig. S15. All samples gained mass over 600-800°C, a result of carbonate formation, also observed with fresh CuO/$SrFeO_3$, presented in Fig. S16. The Cu/$SrFeO_3$ sample gained significantly more mass than either $SrFeO_3$ or Ag/$SrFeO_3$. The mass change between the start and end of the first cycle was greatest again with Cu/$SrFeO_3$, indicating the largest removal of impurities, likely coke in this instance – corroborating the observations in Fig. 10 in the main manuscript.

The first cycle in air for both $SrFeO_3$ and $Ag/SrFeO_3$ showed very additional mass loss compared to the second cycle with most impurities being removed during the cycles in CO_2. Little to no carbonates remained following the cycles in CO_2, as $SrCO_3$ only begins to decompose at >750°C, which was not observed in the first cycle in air. Thus, $SrFeO_3$ and $Ag/SrFeO_3$ were resistant to carbonate reformation in a CO_2 atmosphere (0.33 bar_{CO_2}). The $Cu/SrFeO_3$ sample showed significant additional mass loss at ~700°C compared to the second air cycle. Thus $Cu/SrFeO_3$ appears to be the most prone to carbonate formation in a CO_2 atmosphere. The presence of water in a CO_2 atmosphere has been found to enhance carbonate formation, thus resistance to carbonate formation in a water-free CO_2 atmosphere may not be a reliable indicator for carbonate resistance under dehydrogenation conditions[5]

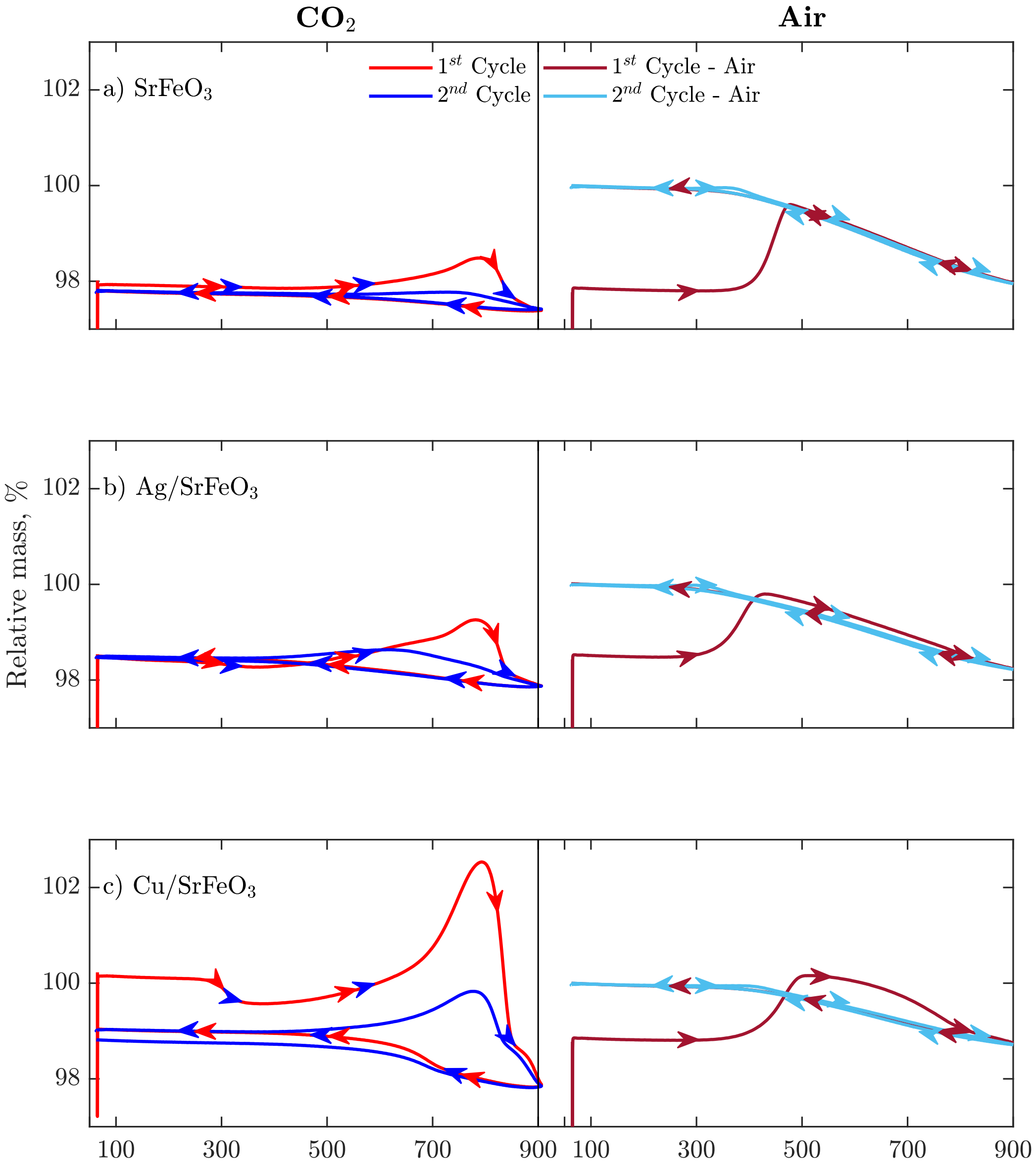

Figure S15: Relative mass changes of spent (a) $SrFeO_3$, (b) $Ag/SrFeO_3$, and (c) $Cu/SrFeO_3$ during 2 cycles in CO_2 followed by 2 cycles in air with temperature cycling between 50 - 900°C and heating rate of 10°C min^{-1}.

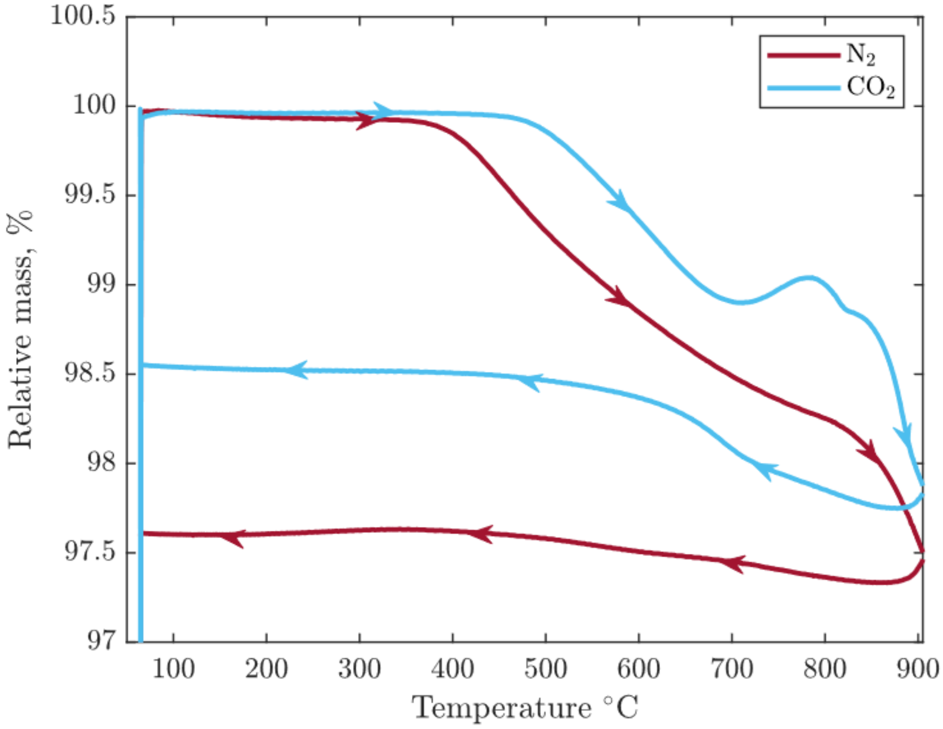

Figure S16: Relative mass change of a sample of fully oxidised and impurity free CuO/$SrFeO_3$ during a temperature-programmed cycle from 50-900°C with a temperature ramp rate of 10°C min^{-1} performed with either N_2 or CO_2 as the reactive gas.

9. Raman analysis of spent and fresh samples

The fresh and spent samples of $SrFeO_3$, CuO/$SrFeO_3$ and Ag/$SrFeO_3$ were characterised using Raman spectroscopy to detect the formation of coke on the surface of the samples. The Raman spectrometer (Horiba Jobin Yvon), equipped with an Olympus BX41 microscope (×50 objective) was used to acquire the spectra of the samples. Raman spectra were excited by a Nd:TAG laser (532.8 nm, 5 mW) in the range of 100 – 1700 cm^{-1}. An exposure time of 30 s was used. To improve the signal-to-noise ratio, the acquisition was repeated five times. Prior to the measurement, the instrument was calibrated using the 520.5 cm^{-1} line of silicon. All spectra were normalised against the maximum intensity of the signal recorded during each scan for a given sample and the results are presented in Fig. S17.

The acquired spectra were compared with the spectra from the literature for $SrFeO_{3-\delta}$[6,7], $SrCO_3$[8] and Ag[9]. For amorphous carbon, Raman responses at ~1350 cm^{-1} (D band) and ~1580 cm^{-1} (G band) were expected[10]. In the analysed samples, the carbon D-band overlapped with the broad spectra of $SrFeO_3$; hence, the presence of coke could not be inferred from the D-band.

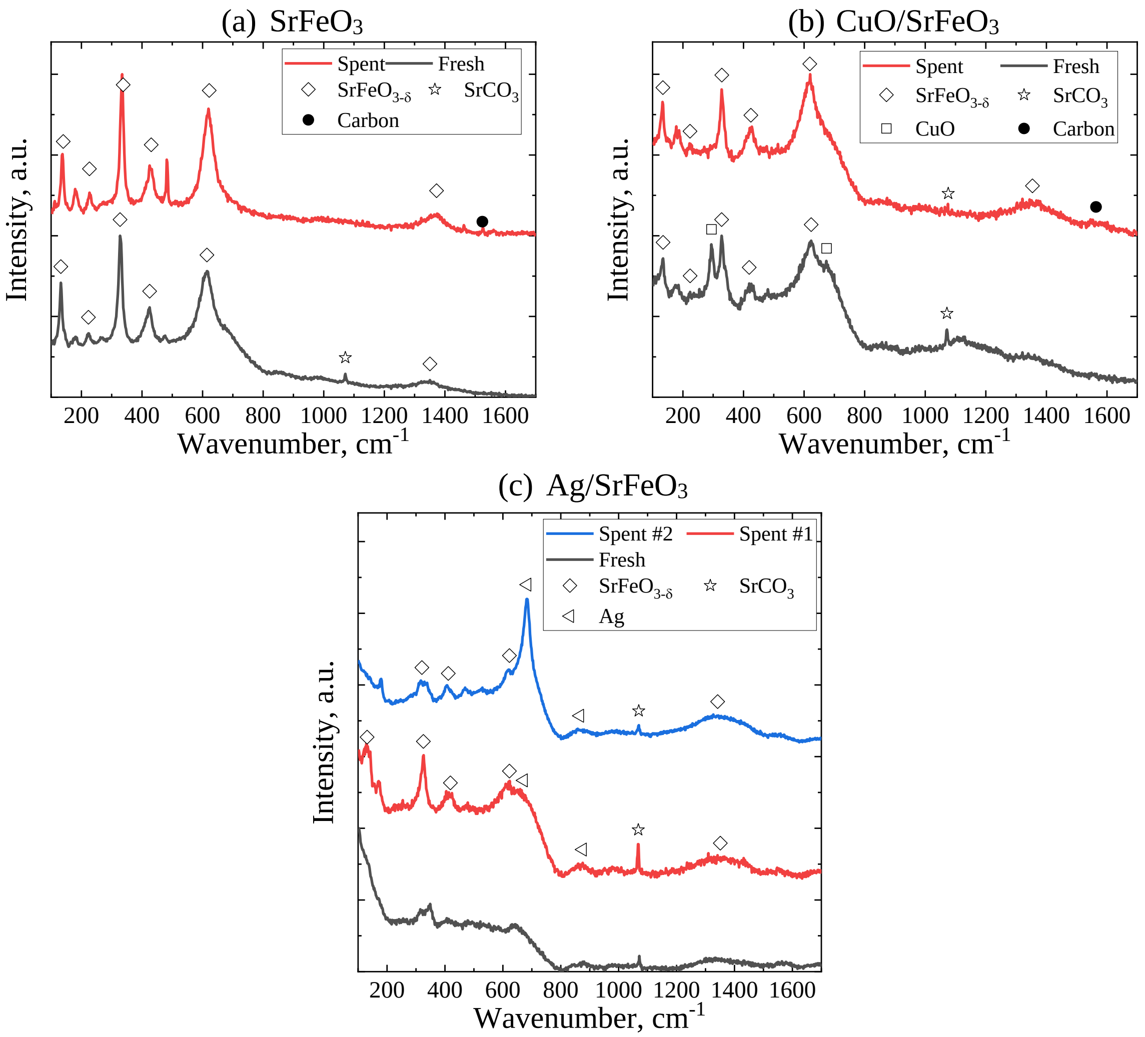

Figure S17: Raman spectra of (a) $SrFeO_3$, (b) $CuO/SrFeO_3$, and (c) $Ag/SrFeO_3$ as fresh and spent materials after reduction in ethanol without reoxidation.

From Fig. S17 (a), $SrCO_3$ was detected in the fresh sample of $SrFeO_3$ but not in the spent samples, after reduction. However, $SrCO_3$ was present in both the fresh and spent samples of $CuO/SrFeO_3$ and $Ag/SrFeO_3$. The intensity of the broad peak at ~ 1370 cm^{-1} for all the spent samples was greater than for the fresh sample, owing to the lower value of oxygen non-stoichiometry, $3 - \delta$, consistent with the findings in the literature[7]. For the spent sample of $Ag/SrFeO_{3-\delta}$, the measurement of Raman spectra was repeated at 50 different locations. Two types of spectra with different relative intensities at various Raman shifts were obtained, presented as Spent#1 and Spent#2. Raman spectra of Spent#1 were slightly more probable, acquired at ~ 30 different locations. The most distinctive difference in Spent#1 and Spent#2 is the intensity at wavenumber of ~680 cm^{-1}, which was attributed to the Ag particles.

In summary, Raman spectra, presented in Fig. S17, showed only very weak evidence of the presence of coke on the surface of samples, $SrFeO_3$ and Cu/ $SrFeO_3$. The inability of Raman spectroscopy to detect coke was not surprising considering the low amount of coke on the samples as shown in the main manuscript, Fig. 10.

10. References

(1) Narayanan, C. R.; Srinivasan, S.; Datye, A. K.; Gorte, R.; Biaglow, A. The Effect of Alumina Structure on Surface Sites for Alcohol Dehydration. Journal of Catalysis 1992, 138 (2), 659–674. https://doi.org/10.1016/0021-9517(92)90314-8.
(2) Marek, E.; Hu, W.; Gaultois, M.; Grey, C. P.; Scott, S. A. The Use of Strontium Ferrite in Chemical Looping Systems. Applied Energy 2018, 223, 369–382. https://doi.org/10.1016/j.apenergy.2018.04.090.
(3) André, L.; Abanades, S. Evaluation and Performances Comparison of Calcium, Strontium and Barium Carbonates during Calcination/Carbonation Reactions for Solar Thermochemical Energy Storage. Journal of Energy Storage 2017, 13, 193–205. https://doi.org/10.1016/j.est.2017.07.014.
(4) Tomita, A. Catalysis of Carbon–Gas Reactions. 8.
(5) Kaus, I.; Wiik, K.; Krogh, B.; Dahle, M.; Hofstad, K. H.; Aasland, S. Stability of SrFeO 3 -Based Materials in H 2 O/CO 2 -Containing Atmospheres at High Temperatures and Pressures. J American Ceramic Society 2007, 90 (7), 2226–2230. https://doi.org/10.1111/j.1551-2916.2007.01727.x.
(6) Radheep, D. M.; Shanmugapriya, • K; Palanivel, • Balan; Ramaswamy Murugan, •. Magnetic Field-Induced Switching of Magnetic Ordering in SrFeO 32d. https://doi.org/10.1007/s00339-016-0303-5.
(7) Barkalov, O. I.; Zaitsev, S. V.; Sedykh, V. D. Strontium Ferrite SrFeO3-δ (2.50 ≤ 3-δ ≤ 2.87) Studied by Raman and Mössbauer Spectroscopy. Solid State Communications 2022, 354, 114912. https://doi.org/10.1016/J.SSC.2022.114912.
(8) Lin, C. C.; Liu, L. G. Post-aragonite phase transitions in strontianite and cerussite—a high-pressure raman spectroscopic study. Journal of Physics and Chemistry of Solids 1997, 58 (6), 977–987. https://doi.org/10.1016/S0022-3697(96)00201-6.
(9) Alzahrani, H. A.; Bravo-Suárez, J. J. In Situ Raman Spectroscopy Study of Silver Particle Size Effects on Unpromoted Ag/α-Al2O3 during Ethylene Epoxidation with Molecular Oxygen. Journal of Catalysis 2023, 418, 225–236. https://doi.org/10.1016/J.JCAT.2023.01.016.
(10) Ferrari, A. C. Raman Spectroscopy of Graphene and Graphite: Disorder, Electron-Phonon Coupling, Doping and Nonadiabatic Effects. Solid State Communications 2007, 143 (1–2), 47–57. https://doi.org/10.1016/J.SSC.2007.03.052.
